# Divergence Times and the Evolutionary Radiation of New World Monkeys (Platyrrhini, Primates): An Analysis of Fossil and Molecular Data

**DOI:** 10.1371/journal.pone.0068029

**Published:** 2013-06-27

**Authors:** S. Ivan Perez, Marcelo F. Tejedor, Nelson M. Novo, Leandro Aristide

**Affiliations:** 1 División Antropología at Museo de La Plata, CONICET, La Plata, Buenos Aires Province, Argentina; 2 Centro Nacional Patagónico, CONICET, Puerto Madryn, Chubut Province, Argentina; Australian Museum, Australia

## Abstract

The estimation of phylogenetic relationships and divergence times among a group of organisms is a fundamental first step toward understanding its biological diversification. The time of the most recent or last common ancestor (LCA) of extant platyrrhines is one of the most controversial among scholars of primate evolution. Here we use two molecular based approaches to date the initial divergence of the platyrrhine clade, Bayesian estimations under a relaxed-clock model and substitution rate plus generation time and body size, employing the fossil record and genome datasets. We also explore the robustness of our estimations with respect to changes in topology, fossil constraints and substitution rate, and discuss the implications of our findings for understanding the platyrrhine radiation. Our results suggest that fossil constraints, topology and substitution rate have an important influence on our divergence time estimates. Bayesian estimates using conservative but realistic fossil constraints suggest that the LCA of extant platyrrhines existed at *ca.* 29 Ma, with the 95% confidence limit for the node ranging from 27–31 Ma. The LCA of extant platyrrhine monkeys based on substitution rate corrected by generation time and body size was established between 21–29 Ma. The estimates based on the two approaches used in this study recalibrate the ages of the major platyrrhine clades and corroborate the hypothesis that they constitute very old lineages. These results can help reconcile several controversial points concerning the affinities of key early Miocene fossils that have arisen among paleontologists and molecular systematists. However, they cannot resolve the controversy of whether these fossil species truly belong to the extant lineages or to a stem platyrrhine clade. That question can only be resolved by morphology. Finally, we show that the use of different approaches and well supported fossil information gives a more robust divergence time estimate of a clade.

## Introduction

The estimation of phylogenetic relationships and divergence times among a group of organisms is a fundamental first step toward understanding its biological diversification [1], [2]. Because of the importance of macroevolutionary and macroecological studies for explaining current diversity and the recent development of statistics for evolutionary inference based on time calibrated phylogenies [2], [3], interest in estimating robust phylogenies and divergence times of different clades has grown significantly. Consequently, divergence times have been widely investigated among several key clades. The Order Primates is one of the most widely studied groups [4]–[6].

Among primates, the temporal divergence of the platyrrhine clade is one of the most controversial among scholars. Platyrrhines are a monophyletic group that migrated into South America and evolved in isolation from the Old World primates. Their current biodiversity stands at 100 to 125 extant species and at least 16 genera [7]–[9]. Within South America and the Caribbean, they experienced a broad radiation occupying a large range of ecological niches, resulting in a great variation in morphology and body size [7], [10]. Although the most recent estimations of platyrrhine phylogeny generated topologies that are generally similar – even considering differences in interpreting the position of *Aotus* [11] – the divergence times are a cause of considerable debate [12]–[17]. Moreover, these divergence time estimations have been used to support or contradict different higher order hypotheses which attempt to explain the shape of platyrrhine evolution. For example, Hodgson et al. [15] used mtDNA data and fossil calibrations to support the idea that platyrrhine diversification is characterized by two successive, sister-group radiations [16], the most recent of which is crown Platyrrhini, and to contradict the so called *long lineages hypothesis* of Rosenberger and co-workers [17]–[20], which interprets possibly all platyrrhines, living and extinct, as belonging to a single holophyletic group, and stresses the role of morphological stasis as a deep evolutionary phenomenon. The latter hypothesis considers the oldest records of platyrrhines (certainly those from the early to middle Miocene of Patagonia and Chile and possibly those from the late Oligocene of Bolivia) as part of the crown Platyrrhini, thus phylogenetically related to the lineages of anatomically modern forms. These also include the most diverse collection of platyrrhine fossils from La Venta, Colombia, deposits of middle Miocene age.

The recent studies of divergence times have used two sources of evidence to discuss estimations, the fossil record and molecular sequences [6], [21], [22]. The fossil record is the only direct source of evidence about the existence of an extant lineage during a time period in the past [4], [5] and it relies on establishing the phylogenetic relationships among fossil and extant forms based on morphological similarities. Particularly, the age of the geological formation containing the fossil provides an unobjectionable minimum boundary for the divergence time of the lineage it represents [5]. Molecular distances between DNA or protein sequences obtained from extant species provide indirect estimations of divergence times, based on the substitutions accumulated along the phylogenetic branches during the divergence process. However, the molecular based method also use external sources of information to calibrate the substitution rates within lineages and derive estimates of divergence times of clades in years, generally fossil record and estimates of the substitution rate per generation [5], [6], [21], [22]. Both approaches have intrinsic sources of uncertainty for divergence time estimations. For the approach that uses the fossil record directly to calibrate the molecular divergence, the uncertainty is related to problems of misclassification or dating error of the fossils [23]. In the approach that uses substitution rate per generation there is uncertainty in the estimations of generation time and substitution rates [22].

These two complementary approaches have been not widely explored in studies of platyrrhine evolution, and thus the debate concerning the divergence schedule of this clade persists unabated. Here we used this arsenal of molecular based approaches to examine the problem, employing the whole mtDNA genome and large-scale nuclear sequence data that are now available for several platyrrhine species [15], [24]–[26]. For estimating divergence times we specifically use (1) a Bayesian Markov Chain Monte Carlo (MCMC) method that co-estimate phylogeny and divergence times under a relaxed-clock model [21] employing multiple fossil constraints and several topological hypotheses, and (2) an alternative approach that is independent of the fossil constraints and employs body size, substitution rate per generation and generation time estimates [22], [27]. We also explore the robustness of our estimations to change in several prior parameters and discuss the implications of our findings for understanding the platyrrhine evolutionary radiation. Specifically, we asked whether the molecular data, fossil constraints and substitution rate information are enough to confidently reject the hypothesis that crown Platyrrhini and/or the main platyrrhine lineages could have diverged at or before 20 Ma (megannum or million years ago). This would constitute a rejection of the long lineage hypothesis, which was supported by Hogdson et al. [15] in a recent influential molecular study.

### South American Land Mammal Ages and the Fossils of the Basal Platyrrhine Radiation

**Figure 1:**
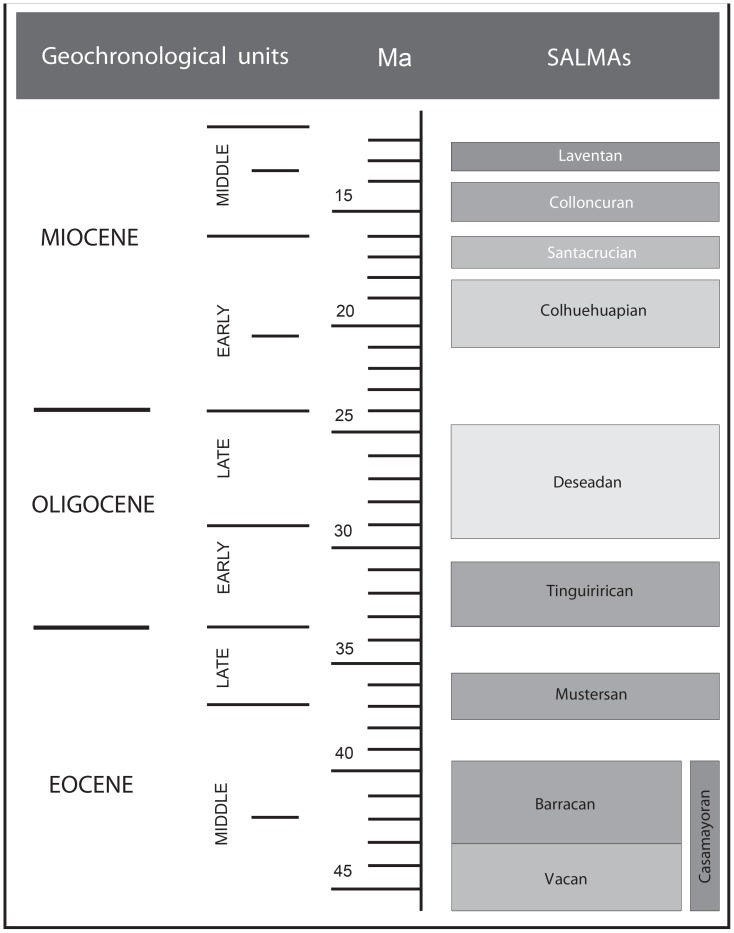
Geologic time scale. Geochronological units and South American Land Mammal Ages (SALMAs) used in the text.

As above mentioned, since the fossil record is the only direct source of evidence about the existence of an extant lineage during a time period in the past, we employ the South American fossil record of mammals, including primates, to establish minimum ages and calibrate phylogenetic trees. That record of mammals is rich in Patagonia, and especially in the primate containing formations, thus allowing correlations with other fossiliferous exposures in South America [20], [28]–[30]. To estimate the divergence times of the New World monkeys, we compiled information (presence/absence of platyrrhine fossils and body size estimations) of eight South American Land Mammal Ages (SALMAs), from the base of the Barrancan subage of the Casamayoran SALMA (41.6 Ma) to the Laventan SALMA (13.8 to 11.8 Ma). We summarized all these biochronological units starting with the oldest records of caviomorph rodents (Figure 1). From the eight SALMAs, primates are absent in the record from the Barrancan (middle Eocene) through the Tinguirirican (early Oligocene; see below), but rodents are relatively well represented by several extinct species.

Except for the Mustersan, three of the oldest SALMAs discussed in this report preserved fossil caviomorph rodents, but no evidence of platyrrhine primates have yet appeared [31]–[37]. By Deseadan times (Figure 1), the oldest records of South American primates, *Branisella* and *Szalatavus*, were present in Salla, Bolivia, dating to the *Branisella*-zone fossiliferous level of about 26 Ma, although the generic status of *Szalatavus* is still debated (see [38]). These primates are known from dental and gnathic anatomical parts that exhibit a mix of unusual characteristics while other traits suggest a close relationship to cebids. Like callitrichines, they have subtriangular upper molars, relatively “waisted” upper premolars, and a conical, *Callimico*-like p2, and a V-shaped mandible. However, they also share unexpected traits such as high-crowned lower molars, and heavy wear on the occlusal surfaces, as well as very small canines. These primates have characteristics that may anticipate the Cebidae, but further studies and more material are needed for a more confident assessment. The estimated body size for these genera was established between 550 and 1000 grams [7]. After these scarce and isolated fossil primates, there is a gap of at least 6 million years with no records of fossil primates. In contrast, and following the previous radiations, Deseadan caviomorphs are abundant and diverse [39].

It is not until the Colhuehuapian SALMA (early Miocene; Figure 1), that primates reappear in Patagonia, Argentina. With 20 Ma *Dolichocebus gaimanensis* [16], [40], [41], from the locality of Gaiman, in Chubut Province, is represented by an edentulous skull and isolated teeth, and is recognized as possibly the earliest known cebine (see [28], [42]; but see [16]) on the basis of characters such as a relatively narrow interorbital septum, a relatively vaulted braincase, as well as oval, vertically oriented orbits and a narrow face. Also, dental traits show similarities to the Laventan *Neosamiri* and *Laventiana*, and by extension to *Saimiri*. But perhaps its most emblematic character, although controversial, is the presence of an interorbital fenestra that appears elsewhere only in the living *Saimiri* [42], thus strengthening their possible phylogenetic relationships. The estimated body size for this genus was established in 2700 grams [7]. Also Colhuehuapian in age, with 20 Ma *Tremacebus harringtoni,* from Chubut Province, Argentina, is recognized as the earliest known aotine based on cranial characters, especially its relatively large orbits, strong postorbital constriction, and short and abbreviated face [28], [43], with a body size estimated in 1800 grams [7]. The morphological debate for the assignment of *Dolichocebus* and *Tremacebus* to the Cebinae and Aotinae clades, respectively, will be dealt with in the Results and Discussion section. Also for the Colhuehuapian the recent description of *Mazzonicebus almendrae*, from Gran Barranca, in south-central Chubut Province [44], expands the record of pitheciines back to the early Miocene (see Results and Discussion) and reinforces the hypothesis of an ancient divergence of living clades. Pitheciines, living and extinct, are characterized mainly by their novel anterior dentition, which is distinctive in their adaptations toward the sclerocarpic harvesting [10], [45]. Another Colhuehuapian genus is *Chilecebus* [28], an unusual primate recovered in central Chile and known from a skull preserving the upper dentition. It shows some characters not found in other platyrrhines, including proportionally large teeth compared to the limited size of the palate. However, the transversely elongated, oval upper premolars are strongly reminiscent of a cebine.

The Santacrucian SALMA (Figure 1) presents a relatively abundant and diverse primate assemblage in Patagonia, and the slightly older Pinturas Formation has yielded four species of primates included in two genera. A third Pinturan genus is being described as well by two of us (MFT and NMN). At about 17 Ma [46], [47], these primates are members of the Pitheciidae, with at least two species of pitheciines allocated to *Soriacebus*. Both exhibit a derived anterior dentition resembling *Mazzonicebus.* There is another group of pitheciids in the Santacrucian as well. From Pinturas, *Carlocebus carmenensis* and *C. intermedius* represent generalized homunculines having close phylogenetic relationships with the younger *Homunculus*, from the Santa Cruz Formation in the southeastern coast of Santa Cruz province (late-early Miocene, 16.5 Ma). These pitheciid genera have body sizes estimated between 2000 and 2700 grams [7]. Finally, also from the Santa Cruz Formation, *Killikaike blakei* was recognized as a cebine closely related to the *Saimiri* lineage [48], mainly based on the morphology of the face (oval orbits vertically oriented, narrow interorbital septum, vaulted frontal bone with a relative anterior brain volume closer to the mean for *Saimiri*).

Thus far, the youngest primate record in Patagonia is *Proteropithecia*, from the Colloncuran SALMA (15.8 Ma; middle Miocene) of Neuquen Province [49]. This primate represents the only uncontroversial pitheciine from the southern regions. All agree it is a member of the crown Platyrrhini.

Far to the northern Neotropics, the middle Miocene primate fauna from La Venta, Colombia, represents the most diverse platyrrhine assemblage in South America and the Caribbean yet discovered [50], [51], as part of the Laventan SALMA (13.8 to 11.8 Ma; middle Miocene; [52]; Figure 1). The Laventan is not represented outside of Colombia, but some possible correlations have been suggested in Bolivia and Patagonia that are still under debate (Croft Quebrada Honda; Chubut Cerro Zeballos). There are 11 described primate species and most of them can be related to living clades, thus representing an uncontroversial cross section of the crown Platyrrhini at about 12.5 Ma. *Neosaimiri fieldsi* and *Laventiana annectens* are certainly cebines pertaining to the *Saimiri* lineage, based on their absolute dental and mandibular similarities to the living squirrel monkey [53]–[55]. *Aotus dindensis* is recognized as an extinct species of *Aotus* for the almost identical mandibular and dental morphology [56]. *Mohanamico hershkovitzi* [57] is probably most closely related to the callitrichines, possibly to the *Callimico* clade [58], based especially on its taller incisors and canines, large p2, and broader and longer trigonid in proportion to the talonid, all callitrichine characters [28]. *Stirtonia tatacoensis* and *S. victoriae* are known by several teeth, a mandible and a maxilla that closely resemble, and are almost indistinguishable from, the living *Alouatta* [53], [59]–[61]. These genera are characterized by a large body size reaching an estimated 10,000 grams [7]. Also with close affinities to a living group, this time with pitheciines, *Cebupithecia sarmientoi* [53] and *Nuciruptor rubricae* [62] exhibit a pitheciine-like molar relief, with low cusps and poorly develped crests, procumbent incisors and projecting canines (excepting *Nuciruptor*), and posteriorly deep mandibles. *Miocallicebus villaviejai* is poorly represented by a piece of maxilla with eroded molars [63], but it seems morphologically close to the living *Callicebus*. However, more remains are needed to strengthen this hypothesis. At least three other genera with uncertain affinities have also been described from the Laventan.

Other fossil platyrrhines have been recovered in younger sediments of South America and in the Greater Antilles (see [20], [28], and references therein). The recognition of phenetic similarities shared between some Caribbean primates and those from Patagonia [64] led us to suspect they are representatives of an old phylogenetic lineage within the crown group.

## Materials and Methods

### Molecular Datasets

To estimate the divergence times of the New World monkeys, we analyzed 13 species of platyrrhines, 14 species of catarrhines, and one outgroup (*Tarsius bancanus*; Table 1). These species were selected (a) in order to provide nodes temporally constrained by well-supported fossil dates and molecular rates [4]–[6], [22]; (b) to take advantage of existing molecular rate estimations for catarrhine primates; and (c) because there are molecular genomic data for all 28 species. Two different molecular datasets were obtained. The first dataset was downloaded from GenBank and is composed of protein mtDNA sequences comprising a 12,996 bp matrix (Table 1). These mtDNA sequences were aligned using ClustalW. The alignment was in the reading frame and examined for ambiguous regions with BioEdit 7.0.0 software [65]. The dataset did not have important ambiguous regions. The second dataset was obtained from the supporting information in the Perelman *et al.* [26] study. This dataset is a post-GBLOCK editing alignment including 54 coding and non-coding nuclear sequences and comprises a 34,941 bp matrix (Table 1), constituting a representative stratified sample of the whole genome [26].

**Table 1:** Molecular data.

Species	mtDNA sequences	Nuclear sequences
*Tarsius bancanus*	NC_002811	Perelman et al. [26]
*Homo sapiens* (Cambridge)	NC_012920	Perelman et al. [26]
*Pan paniscus*	GU189661	Perelman et al. [26]
*Pan troglodytes*	NC_001643	Perelman et al. [26]
*Pan troglodytes verus*	X93335	Perelman et al. [26]
*Gorilla gorilla*	NC_001645	Perelman et al. [26]
*Pongo pygmaeus*	NC_001646	Perelman et al. [26]
*Hylobates lar*	NC_002082	Perelman et al. [26]
*Macaca mulatta*	NC_005943	Perelman et al. [26]
*Macaca sylvanus*	NC_002764	Perelman et al. [26]
*Papio hamadryas*	NC_001992	Perelman et al. [26]
*Theropithecus gelada*	FJ785426	Perelman et al. [26]
*Cercopithecus aethiops*	AY863426	Perelman et al. [26]
*Chlorocebus sabaeus*	EF597503	Perelman et al. [26]
*Colobus guereza*	AY863427	Perelman et al. [26]
*Callithrix jacchus*	AB572419	Perelman et al. [26]
*Saguinus oedipus*	FJ785424	Perelman et al. [26]
*Cebus apella*	JN380205	Perelman et al. [26]
*Cebus albifrons*	AJ309866	Perelman et al. [26]
*Saimiri boliviensis boliviensis*	HQ644339	Perelman et al. [26]
*Saimiri oerstedii oerstedii*	HQ644337	Perelman et al. [26]
*Saimiri sciureus*	FJ785425	Perelman et al. [26]
*Aotus azarai azarai*	JN161099	Perelman et al. [26]
*Aotus lemurinus*	FJ785421	Perelman et al. [26]
*Aotus nancymaae*	JN161101	Perelman et al. [26]
*Aotus trivirgatus*	AY250707	Perelman et al. [26]
*Ateles belzebuth*	FJ785422	Perelman et al. [26]
*Callicebus donacophilus*	FJ785423	Perelman et al. [26]

List of species used in the study and Genbank accession numbers.

### Phylogenetic Tree and Divergence Time Estimations

The best-fitting model of evolution for each sequence studied was estimated employing the Akaike information criterion with correction for sample size (AICc) implemented in jModelTest 0.1.1 [66]. The results are shown in table 2. Models of sequence evolution identified as optimal by jModelTest for both coding and non-coding sequences were implemented in the phylogenetic analyses.

**Table 2:** Substitution models.

Sequence	nst	rates	model	Sequence size
ABCA1	2	gamma	HKY+G	560
ADORA3	2	gamma	HKY+G	414
AFF2	6	gamma	GTR+G	500
AFF2.2	6	gamma	GTR+G	579
APP	6	gamma	GTR+G	672
AXIN1	6	gamma	HKY+I	949
BCOR	6	gamma	GTR+G	771
BDNF	2	gamma	HKY+G	561
BRCA2	6	gamma	GTR+G	1252
CFTR	2	gamma	HKY+G	791
CHRNA1	2	gamma	GTR+G	381
CNR1	2	gamma	HKY+I+G	998
CREM	2	gamma	HKY+G	428
DACH1	2	gamma	HKY+I+G	627
DMRT1	2	gamma	HKY+G	537
EDG1	2	gamma	HKY+G	967
FBN1	2	gamma	HKY+G	720
FES	2	gamma	HKY+G	469
FOXP1	6	gamma	GTR+G	564
GHR	2	gamma	HKY+G	646
KCNMA1	6	gamma	GTR+G	614
LRPPRC_169	2	gamma	HKY+G	792
LRPPRC_171	6	gamma	GTR+G	761
LUC7L	6	gamma	GTR+G	694
MAPKAP1	6	gamma	GTR+G	655
MBD5	2	gamma	HKY+I+G	558
NEGR1	6	gamma	GTR+G	540
NPAS3	6	gamma	GTR+G	605
NPAS3.2	6	gamma	GTR+G	650
PLCB4	6	gamma	GTR+G	338
RAG1	2	gamma	HKY+I+G	1071
RAG2	6	gamma	GTR+G	690
RPGRIP1	2	gamma	HKY+I+G	683
SGMS1	1	gamma	HKY+G	598
SIM1	2	gamma	HKY+G	646
SMCX	6	gamma	GTR+G	330
SMCY	2	gamma	HKY+G	940
SRY	2	gamma	HKY+G	467
TEX2	1	equal	HKY	156
TTR	6	gamma	GTR+G	877
TYR	2	gamma	HKY+G	475
USH2A	6	gamma	GTR+G	605
UTY	2	equal	GTR	371
ZFX	6	gamma	GTR+G	811
ZFY	6	gamma	GTR+G	853
ZIC3	2	equal	HKY	549
ATXN7	2	equal	HKY+I+G	523
BCHE	6	gamma	GTR+G	984
DCTN2	6	gamma	GTR+G	605
FAM123B	2	gamma	HKY+I+G	730
PNOC	2	gamma	HKY+G	313
POLA1	6	gamma	GTR+G	604
RAB6IP1	2	gamma	HKY+I+G	717
ERC2	6	gamma	GTR+G	750
Total nuclear	6	gamma	GTR+I+G	34941
mtDNA	6	gamma	GTR+I+G	12996

Coding and non-coding sequences used in the current study, sequence size (bp) and substitution models.

Two divergence time estimation approaches were used [21], [22], [27]. Firstly, the phylogenetic tree topology and divergence times were estimated jointly using the BEAST v1.6.1 package [21], [67]. We used the BEAUti program to unlink the substitution models of the data partitions and to implement the models of sequence evolution identified as optimal by jModelTest. We analyzed the sequences under a relaxed molecular clock model, which allows substitution rates to vary across branches according to an uncorrelated lognormal distribution [21], and set the species tree priors as a Yule Process. Two simultaneous analyses were performed using Markov Chain Monte Carlo (MCMC) simulations for 200,000,000 generations with a sampling frequency of 20,000. The convergence was determined with Tracer v1.5 [68] and the first 2,500 trees sampled were excluded using TreeAnnotator v1.4.8 [67]. FigTree v1.3.1 was used to plot all phylogenetic trees.

Uncertainty in divergence time estimation using BEAST could be mainly related to uncertainty in tree topology and fossil calibrations [21]. Because there are different hypotheses of topological relationships among the main extant lineages (families and subfamilies) of platyrrhines, as discussed above, we changed the best inferred topology for each dataset by enforcing monophyly constraints on several clades (Table 3). This procedure made the resulting trees consistent with previous studies of platyrrhine phylogeny [10], [11], [13], [26], [69]. We generated 4 alternative tree topologies: (1) Atelidae sister to Cebidae, with Aotinae as a branch external to the Cebidae family [69]; (2) Atelidae sister to Cebidae, with Aotinae as a branch external to the Callitrichinae subfamily [26]; (3) Atelidae sister to Cebidae, with Aotinae being related to Cebinae [13]; and (4) Pitheciidae sister to Atelidae, with Aotinae related to *Callicebus* [10], [18].

**Table 3:** Alternative topologies.

Topologies	Wildman et al [69]	Opazo et al. [13] *	Perelman et al. [26] **	Rosenberger [10], [18]
**Monophyly constrained clades**	Cebinae (*Saimiri*-*Cebus*)	Cebinae (*Saimiri*-*Cebus*)	Cebinae (*Saimiri*-*Cebus*)	Cebinae (*Saimiri*-*Cebus*)
	Cebidae – Aotinae	Cebinae – Aotinae	Aotinae (*Aotus*) – Callitrichinae	Aotinae (*Aotus*) – *Callicebus*
	Cebinae – Callitrichinae	Cebidae – Aotinae	Cebidae – Aotinae	Pitheciidae (Aotinae-*Callicebus*) – Atelidae
	Cebidae-Aotinae-Atelidae	Cebidae-Aotinae-Atelidae	Cebidae-Aotinae-Atelidae	Cebinae – Callitrichinae

Monophyly constraints on platyrrhine clades.

* Best inferred topology for mtDNA. Topology inferred without monophyly constraints.

** Best inferred topology for nuclear data. Topology inferred without monophyly constraints.

**Figure 2:**
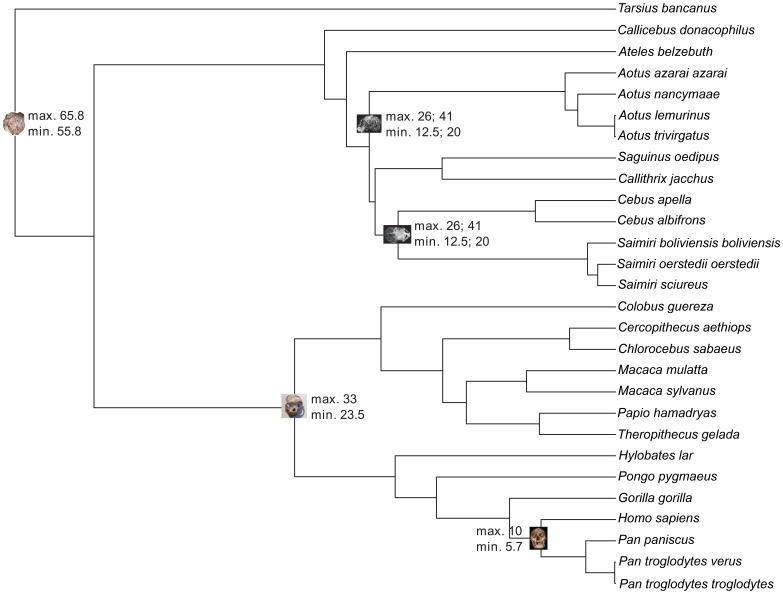
Fossil calibrations. Phylogenetic tree of 28 primate species showing fossil calibrations. Calibration bounds are soft; i.e., the probability that the true divergence time is outside the bounds is small but non-zero [6]. The phylogenetic tree follows the Wildman et al. [69] hypothesis.

For the four topologies, five fossil calibrations were selected based on criteria for choosing appropriate points [4], [6], two nodes each for the platyrrhine and catarrhine clades and one for the outgroup. Both minimum and maximum calibration bounds were set to the probability that the true divergence time is outside the bounds to be small, but non-zero (i.e., ‘soft’ for [6]). Fossil calibration for catarrhines was obtained from Benton et al. [4] and Steiper and Seiffert ([27]; Figure 2; Table 4). Because phylogenetic interpretations of the fossil record of platyrrhines is still debated [16], [17], [28], [70], we also set three fossil calibration hypotheses for the two nodes (Figure 2; Table 4). Our approach was designed to estimate the time of origin of the crown Platyrrhini without using any specific fossil-based calibration constraint for this particular node of the molecular tree; however, in the first and second hypotheses, *Dolichocebus* and *Tremacebus,* whose membership to the platyrrhine crown is contentious, are used as calibration points for extant families (see next and Results and Discussion). The first hypothesis is based on the most traditional phylogenetic interpretation for Patagonian fossils [17], [28]: (1) minimum divergence time of Cebinae was set at 20 Ma, based on *Dolichocebus gaimanensis*, a fossil from the valley of the Chubut river in Argentina, attributable to Cebinae; maximum divergence time of Cebinae was set at 26 Ma, based on the absence of Cebinae fossils previous to the Deseadan fauna of Salla, Bolivia; (2) minimum divergence time between Aotinae and Cebinae, Callitrichinae or *Callicebus* (depending on the topology) was set at 20 Ma, based on *Tremacebus harringtoni*, a fossil from Sacanana, Chubut Province in Argentina, attributable to Aotinae; maximum divergence time between Aotinae and Cebinae, Callitrichinae or *Callicebus* was set at 26 Ma, based on the absence of aotine fossils in the Deseadan fauna of Salla, Bolivia, and other South American formations of the same age. The second hypothesis is a modification of the first one based on an alternative phylogenetic interpretation for Patagonian fossils [15], [16], [70]. We modified the minimum divergence time of Cebinae and Aotinae using a calibration at 12.5 Ma, based on *Neosaimiri fieldsi* and *Aotus dindensis*, respectively, two fossil species from La Venta, in Colombia, which we recognize (and is now apparently the consensus view among active researchers) as cebines and aotines, respectively. The third hypothesis is a modification of the first one based on an alternative soft maximum divergence time of Cebinae and Aotinae using a calibration at 41 Ma, based on the absence of Cebinae and Aotinae fossils in the Contamana fauna, Peru, and other ancient South American formations of the same age [31]. All calibration points were implemented as log-normal distributions with an offset, mean, and standard deviation such that 95% of the prior distribution falls between the boundaries specified in figure 2 and table 4. This procedure allows molecular data to correct for conflicting fossil information and uncertainty in the in fossil evidence [6], [21].

**Table 4:** Calibrations.

		Node	Minimum soft bound	Fossilminimum	Maximum soft bound	Evidence maximum	Parameters of the LogNormal distribution
Old World Primates		Root Haplorhini	55.80	*Teilhardina*	65.80	absence of euprimates in Paleocene	Offset (55.8), mean (1.3), and standard deviation (0.5), Initial Value = 60
		Catarrhini	23.50	*Proconsul*	33.00	absence of hominoids in late Eocene	Offset (23.5), mean (1.5), and standard deviation (0.5)
		Hominini	5.70	*Orrorin*	10.00	absence of hominines in mid-Miocene	Offset (5.7), mean (0.5), and standard deviation (0.5)
New World Primates	**First hypothesis** *	Cebinae (*Saimiri*-*Cebus*)	20.00	*Dolichocebus*	26.00	absence of cebids after the Salla formation	Offset (20), mean (0.9), and standard deviation (0.4)
		Aotinae/−	20.00	*Tremacebus*	26.00	absence of aotines after the Salla formation	Offset (20), mean (0.9), and standard deviation (0.4)
	**Second hypothesis** **	Cebinae (*Saimiri*-*Cebus*)	12.50	*Neosaimiri*	26.00	absence of cebids after the Salla formation	Offset (12.5), mean (1.8), and standard deviation (0.4)
		Aotinae/−	12.50	*Aotus dindensis*	26.00	absence of aotines after the Salla formation	Offset (12.5), mean (1.8), and standard deviation (0.4)
	**Third** **hypothesis** ***	Cebinae (*Saimiri*-*Cebus*)	20.00	*Dolichocebus*	41.00	absence of cebids after Contamana	Offset (20), mean (2), and standard deviation (0.5)
		Aotinae/−	20.00	*Tremacebus*	41.00	absence of aotines after Contamana formation	Offset (20), mean (2), and standard deviation (0.5)

Fossil calibrations and prior distribution values.

* Fleagle and Tejedor [89], Rosenberger [17];

** Kay et al. [16];

*** Antoine et al. [31].

Secondly, for comparative purposes we combined and applied a slight modification of the methods recently proposed by Steiper and Seiffert [27] and Langergraber et al. [22]. This method is based on the argument that by estimating an external molecular rate, or the rate at which the DNA sequence diverged in the genome, DNA differences can be converted into divergence times independently of fossil calibration constraints [22], [71]. The only available direct estimation of a molecular rate among primates is for the human lineage [22]. Since this rate is not necessarily the same for platyrrhines, a procedure to correct for possible differences is needed. The rationale behind the method proposed here is based on the Steiper and Seiffert work [27], which showed that there is a strong, inverse relationship between molecular substitution rates and body size for primates. It is also known that body size is correlated with primate life history (e.g. generation time) [72]. The relationship between generation time and substitution rate is based on the hypothesis that most germ-line mutations occur during DNA replication [73]. The obtained correlation coefficient between body mass and generation time is 0.89 (P>0.001) for extant platyrrhines and 0.907 (P>0.001) for all primates used in this study (see Material S1 for details). Using this, we employed body size and generation time estimates for extant and extinct platyrrhines to obtain a corrected substitution rate that is applicable to the different platyrrhine lineages, based on an estimation independent from the fossil constraint. This alternative method was only used with the nuclear DNA dataset (following Langergraber et al. [22]), since mitochondrial substitution rates are known to differ from nuclear ones and a time-dependent rate-curve effect is observed in mitochondrial DNA.

Uncertainty in divergence time estimations using an external molecular rate could be mainly related to uncertainty in substitution rate and generation time estimations. As in Langergraber et al. [22] we used the broadest available interval of substitution rates estimations –based on human mutation rates– to incorporate the first source of uncertainty in our analyses (9.70E-09 to 1.36E-08/site/generation [22]). Because a substitution rate independent from the fossil record is only available for a single species (*Homo sapiens*), we applied three imputation procedures to infer generation time and correct the substitution rate: a linear regression, a quadratic curve and the EM algorithm ([74]; see Material S1 for details). After careful inspection of the resulting imputation, we used the quadratic curve results in the following analyses (see Material S1; Table 5). We used the mean generation time inferred for each clade as the best estimation of generation times along their whole evolutionary history. This is a different approach to that of Steiper and Seiffert [27] (see Results and Discussion). In this way, the changes in substitution rates along the tree are a function of changes in body size and generation time of the studied primate species, as would be predicted by the hypothesis that most mutations occur during DNA replication [73].

**Table 5:** Size and generation time.

Clade	Genus	Body size in grams	Generation time in years
Atelidae	*Alouatta*	6404.2	12.0
Atelidae	*Ateles*	8276.3	15.0
Atelidae	*Brachyteles*	8840.0	20.0
Atelidae	*Lagothrix*	7150.0	15.0
Aotinae	*Aotus*	1018.7	8.0
Cebinae	*Cebus*	2475.1	15.0
Cebinae	*Saimiri*	786.9	8.0
Callitrichinae	*Saguinus*	444.4	6.0
Callitrichinae	*Leontopithecus*	471.4	7.0
Callitrichinae	*Callithrix*	351.2	6.0
Callitrichinae	*Callimico*	505.0	6.0
Pitheciidae	*Callicebus*	997.3	8.0
Pitheciidae	*Pithecia*	2003.5	9.0
Pitheciidae	*Cacajao*	2893.8	10.0
Pitheciidae	*Chiropotes*	2632.5	10.0
Pitheciidae	† *Soriacebus*	2000.0	9.0*
Pitheciidae	† *Carlocebus*	2000.0	9.0*
Pitheciidae	† *Homunculus*	2700.0	10.0*
Pitheciidae	† *Cebupithecia*	2200.0	9.0*
Pitheciidae	† *Nuciruptor*	2000.0	9.0*
Pitheciidae	† *Proteropithecia*	1600.0	9.0*
Aotinae	† *Tremacebus*	1800.0	9.0*
Aotinae	† *Aotus (dindensis)*	1000.0	8.0*
Cebinae	† *Dolichocebus*	2700.0	10.0*
Cebinae	† *Chilecebus*	1000.0	8.0*
Cebinae	† *Neosaimiri*	840.0	8.0*
Cebinae	† *Laventiana*	800.0	8.0*
Atelidae	† *Stirtonia*	5800.0	12.0*
Atelidae	† *Stirtonia*	10000.0	20.0*
Atelidae	† *Protopithecus*	23500.0	22.0*
Atelidae	† *Caipora*	24000.0	22.0*
Callitrichinae	† *Patasola*	1000.0	8.0*
Callitrichinae	† *Lagonimico*	1300.0	8.0*
*Incertae sedis*	† *Branisella*	1000.0	8.0*
*Incertae sedis*	† *Szalatavus*	550.0	7.0*
Hominidae	*Homo*	45000.0	29.0
Hominidae	*Pan*	33000.0	25.0
Hominidae	*Gorilla*	71000.0	19.0
Cercopithecinae	*Macaca*	9000.0	10.0

Adult body size and generation time for extant and fossil genera*.

† Fossil genera.

* Generation time was estimated for fossil genera using the inferred body size [7]. Body size for extant taxa was obtained from Smith and Jungers [79].

After correcting the substitution rates for each studied hominid and platyrrhine lineage, we estimated divergence time for each node of interest. For this, we estimated a Maximum Likelihood tree with a general time reversible substitution model and gamma distribution and then constructed a linearized tree using Mega 5.05 [75], [76]. For the different branches of this tree we specified the previously estimated different substitution rates. Prior to each calculation, we conducted Tajima’s relative rate test [77], or molecular clock hypothesis test, for the molecular divergence between the two species compared using Mega 5.05 [76]. The test was only significant for comparisons that involved *Aotus*, and therefore this genus was excluded from the analysis. Generation time for extant platyrrhine species was obtained from IUCN [78] and average body mass for wild adults was obtained from Smith and Jungers [79] and for fossils from Fleagle [7].

## Results and Discussion

### Phylogenetic Tree

**Figure 3:**
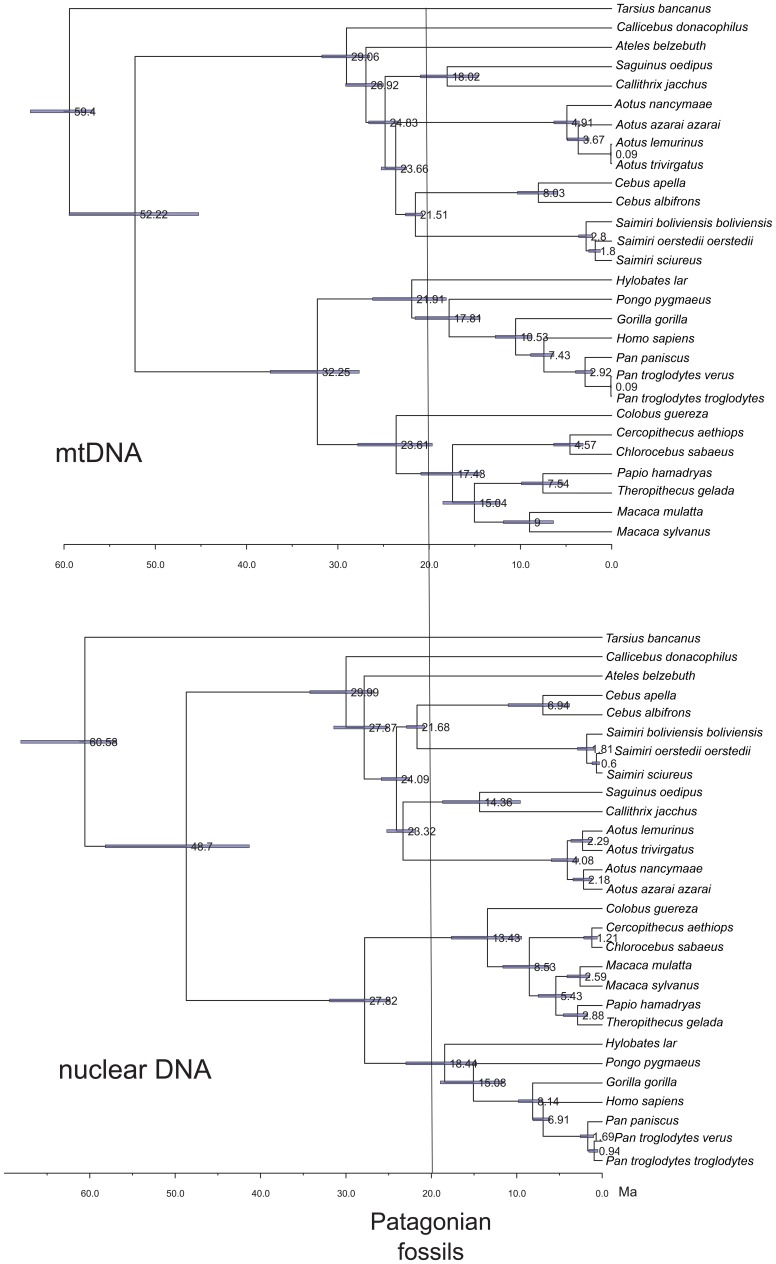
BEAST chronophylogenetic trees. More likely **c**hronophylogenetic tree from the BEAST analysis for 28 species of Primates using mtDNA and nuclear sequences. Mean node ages are depicted in each node. Blue horizontal bars represent the posterior 95% CI for the node ages. The vertical line shows the estimated earliest age of Patagonian lineages.

We used the complete set of the protein-encoding genes from the mitochondrial genome and a large-scale stratified sampling of coding and non-coding nuclear sequences from several species taken from GenBank to represent the major platyrrhine lineages. We also used DNA data from several other haplorhine species with a well known fossil record to provide nodes that are temporally constrained (Table 4). Our chronophylogenetic trees based on these mtDNA and nuclear data with their maximum likelihood values are in agreement with other recent molecular trees (Figure 3), which support the division of platyrrhines into three monophyletic families (Atelidae, Cebidae, and Pitheciidae) and suggest a sister-group phylogenetic relationship between Atelidae and Cebidae [11], [13], [26], [69]. Within the family Cebidae, these trees display a branch for Cebinae, which includes *Cebus* and *Saimiri*, as well as a branch for Callitrichinae formed by *Saguinus* and *Callithrix*. The relationships among Old World monkeys are also in agreement with recent phylogenies [26]. These trees only differ in the *Aotus* position; it is phylogenetically related to Callitrichinae for the nuclear dataset, as per the Perelman et al. [26] tree, and to Cebinae for the mtDNA dataset, as in the Opazo et al. [13] tree, but both positions occur with low node support.

The alternative chronophylogenetic trees with constrained topologies based on previous molecular tree hypotheses display maximum likelihood values that are not significantly different from Perelman et al. [26] and Opazo et al. [13] for nuclear and mtDNA datasets, respectively (Table 6). This is not surprising given the existence of short branch lengths connecting Cebinae, Callitrichinae and Aotinae lineages (Figure 3) and the previous problems in estimating a robust platyrrhine species tree [11], [80]. On the other hand, the chronophylogenetic trees with a constrained topology based on the Rosenberger [10] morphological hypothesis display maximum likelihood values that are significantly lower than the Perelman et al. [26] and the Opazo et al. [13] trees (Table 6). We do not know whether one of these inferred phylogenetic trees is representative of the true branching process or history of platyrrhine species divergence. Although previous studies suggest that this is not problematic for divergence time estimation, our results suggest that the topology has great importance for inferring the divergence time of the main platyrrhine lineages (Table 7). For this reason we considered the alternative topologies in divergence time estimates that are discussed in the following section.

**Table 6:** Likelihood values.

Dataset	Topology	Likelihood mean	Likelihood median	95% HPD lower	95% HPD upper
**mtDNA**	Opazo	**−129818.64**	**−129818.30**	**−129829.13**	**−129807.70**
	Perelman	**−** *129824.11*	**−** *129823.68*	**−** *129835.01*	**−** *129813.66*
	Wildman	**−** *129831.34*	**−** *129831.05*	**−** *129842.25*	**−** *129820.89*
	Rosenberger	**−**129883.51	**−**129883.15	**−**129894.68	**−**129872.73
**Nuclear DNA**	Perelman	**−100064.87**	**−100064.51**	**−100076.67**	**−100053.04**
	Wildman	**−** *100068.80*	**−** *100068.46*	**−** *100080.92*	**−** *100057.59*
	Opazo	**−** *100073.10*	**−** *100072.69*	**−** *100085.33*	**−** *100061.60*
	Rosenberger	**−**100292.04	**−**100291.69	**−**100303.89	**−**100279.85

Likelihood for alternative BEAST topologies. The most likely tree is displayed in bold.

**Table 7:** Bayesian divergence time estimations.

Dataset	Approach	Node	Topology Wildman	Topology Perelman	Topology Opazo	Topology Rosenberger
**mtDNA**	**First hypothesis** *	Crown Platyrrhini	**28.78 (26.37–31.50)**	**28.52 (26.12–31.22)**	**29.06 (26.52–31.75)**	**24.99 (23.58–26.68)**
		Atelidae branching	26.61 (24.76–28.73)	26.36 (24.58–28.52)	26.92 (25.02–29.14)	24.19 (22.72–25.99)
		Crown Cebidae	24.35 (22.95–25.94)	24.17 (22.78–25.70)	24.82 (23.39–26.62)	24.34 (22.90–25.95)
		Crown Anthropoidea	51.58 (44.10–58.81)	51.76 (44.57–58.65)	52.22 (45.25–59.44)	50.31 (43.28–57.95)
		Crown Catarrhini	31.74 (27.33–36.78)	32.03 (27.14–36.85)	32.25 (27.66–37.41)	31.43 (27.06–36.28)
		*Homo/Pan*	7.37 (6.33–8.87)	7.31 (6.30–8.62)	7.43 (6.33–8.87)	7.38 (6.36–8.77)
	Second hypothesis**	Crown Platyrrhini	**24.28 (21.22–27.93)**	**23.99 (20.89–27.37)**	**24.37 (21.25–27.70)**	**20.85 (18.45–23.78)**
		Atelidae branching	22.26 (19.55–25.53)	22.02 (19.47–25.15)	22.45 (19.61–25.32)	20.18 (17.68–23.07)
		Crown Cebidae	20.19 (17.79–23.10)	20.02 (17.52–22.64)	20.56 (18.05–23.16)	20.24 (17.79–23.02)
		Crown Anthropoidea	46.95 (39.29–55.49)	46.56 (39.71–55.21)	46.67 (39.47–54.46)	46.29 (39.44–54.27)
		Crown Catarrhini	29.12 (25.62–33.38)	29.07 (25.55–33.08)	29.07 (25.58–33.00)	29.03 (25.69–33.26)
		*Homo/Pan*	7.04 (6.20–8.25)	7.03 (6.19–8.33)	7.07 (6.19–8.29)	7.10 (6.19–8.36)
	Third hypothesis***	Crown Platyrrhini	**31.07 (27.93–34.57)**	**30.74 (27.78–34.49)**	**31.31 (27.89–35.36)**	**27.26 (24.76–30.12)**
		Atelidae branching	28.79 (26.25–31.93)	28.50 (25.98–31.66)	29.11 (26.27–32.48)	26.48 (24.05–29.45)
		Crown Cebidae	26.44 (24.18–29.10)	26.25 (24.06–28.91)	26.92 (24.55–25.75)	26.47 (24.17–29.34)
		Crown Anthropoidea	53.85 (46.14–60.52)	53.87 (45.75–60.53)	54.25 (46.91–60.38)	52.61 (45.08–59.54)
		Crown Catarrhini	33.01 (27.99–38.39)	33.11 (27.64–38.35)	33.52 (28.58–38.88)	32.69 (27.74–38.35)
		*Homo/Pan*	7.49 (6.30–9.14)	7.51 (6.34–8.97)	7.47 (6.30–8.99)	7.45 (6.25–8.88)
**Nuclear**	**First hypothesis** *	Crown Platyrrhini	**30.06 (26.77–33.88)**	**29.99 (26.79–34.22)**	**30.04 (26.91–34.02)**	**25.65 (23.80–27.93)**
		Atelidae branching	27.91 (25.32–31.46)	27.87 (25.16–31.42)	27.87 (25.14–31.28)	25.52 (23.56–27.74)
		Crown Cebidae	24.27 (22.71–26.18)	24.09 (22.55–25.37)	24.04 (22.64–26.03)	23.88 (22.33–25.71)
		Crown Anthropoidea	48.80 (41.82–58.46)	48.70 (41.32–58.19)	48.65 (41.38–57.68)	46.16 (38.66–56.02)
		Crown Catarrhini	27.85 (24.94–32.04)	27.82 (24.82–31.92)	27.77 (25.02–32.82)	27.53 (24.90–31.22)
		*Homo/Pan*	6.91 (6.11–8.04)	6.91 (6.11–8.10)	6.91 (6.11–8.07)	6.91 (6.12–8.09)
	Second hypothesis**	Crown Platyrrhini	**25.57 (21.91–30.06)**	**25.61 (21.72–30.35)**	**25.65 (21.89–30.22)**	**21.89 (18.94–25.31)**
		Atelidae branching	23.58 (20.02–27.33)	23.61 (20.31–27.98)	23.68 (20.12–27.66)	21.79 (18.95–25.31)
		Crown Cebidae	19.97 (17.43–22.92)	19.93 (17.24–22.83)	19.95 (17.51–23.08)	19.79 (17.14–22.76)
		Crown Anthropoidea	44.17 (36.69–53.75)	44.55 (36.56–53.83)	44.49 (36.93–53.67)	43.45 (36.08–53.34)
		Crown Catarrhini	27.12 (24.8–30.41)	27.21 (24.69–30.80)	27.07 (24.58–30.59)	26.98 (24.65–30.36)
		*Homo/Pan*	6.83 (6.13–7.95)	6.81 (6.05–7.88)	6.82 (6.09–7.90)	6.81 (6.09–7.84)
	Third hypothesis***	Crown Platyrrhini	**32.92 (28.60–37.91)**	**32.94 (28.84–38.36)**	**33.06 (28.83–38.41)**	**28.67 (25.81–32.74)**
		Atelidae branching	30.72 (27.06–35.31)	30.69 (27.06–35.61)	30.82 (26.97–35.52)	28.55 (25.60–32.54)
		Crown Cebidae	26.89 (24.22–30.43)	26.78 (24.05–30.13)	26.72 (24.04–30.22)	26.34 (23.73–29.76)
		Crown Anthropoidea	51.04 (42.47–59.59)	51.61 (43.08–60.36)	51.42 (43.41–60.33)	49.04 (41.61–59.15)
		Crown Catarrhini	28.30 (25.06–33.23)	28.14 (25.09–33.00)	28.11 (25.00–32.87)	28.05 (24.94–32.46)
		*Homo/Pan*	6.96 (6.11–8.24)	7.00 (6.10–8.37)	6.98 (6.15–8.35)	6.96 (6.12–8.27)

Posterior means and 95% confidence intervals of divergence time (in millions of years) for selected nodes in alternative platyrrhine tree topologies under different fossil calibrations.

* Fleagle and Tejedor [89], Rosenberger [17]; **Kay et al. [16]; ***Antoine et al. [31].

### Bayesian Divergence Times Estimations under a Relaxed-clock Model

The Bayesian phylogenetic method used in our analyses provide a framework to co-estimate phylogenetic relationships and divergence times under a relaxed molecular clock model [21]. Estimating divergence times using DNA data and fossil calibrations is a complex process as it accounts for fossil age constraints, tree topology and models of molecular evolution. In particular, the fossil calibration points and tree topology must be carefully considered [6], [21] because these parameters can generate very different divergence estimations. Our estimates for the Old World monkeys are in agreement with those obtained in recent studies (Table 7; [5], [6]). Divergence time estimates for platyrrhines based on the different molecular topologies display similar values, but the estimates based on the Rosenberger [10], [18] topology shows values *ca.* 4 Ma younger (Table 7; Figure S1–S4). This result contrasts with previous studies that suggested that differences in tree topology among platyrrhine trees are not problematic for divergence time estimation [6], [15]. Therefore, we confirm previous suggestions that the Bayesian estimation of phylogeny and divergence time from DNA sequences may be biased when the tree topology is not adequately considered in the model [21].

Our divergence time estimates using the more likely topologies and the most conservative fossil evidence (Second Hypothesis) suggest that the last common ancestor (LCA) of extant platyrrhine primates existed at *ca.* 25 Ma, with the 95% confidence limit for the node ranging from *ca.* 21–30 Ma (Table 7; Figure S1–S4). However, the fossil constraints also have an important influence over our divergence time estimates. The results of the divergence estimates using the alternative First and Third Hypotheses show older time values for the LCA than the estimates using the Second Hypothesis. The LCA value was *ca.* 29 Ma for the First Hypothesis (Figure 3) and *ca.* 32 Ma for the Third Hypothesis, with the 95% confidence limit for the node ranged from *ca.* 27–31 Ma and *ca.* 27–34 Ma, respectively (Table 7). The divergence time estimates based on the mtDNA dataset are always *ca.* 1 Ma younger than the ones based on the nuclear dataset (Table 7; Figure S1–S4). For all the hypotheses, our results suggest that the extant platyrrhine families diverged before *ca.* 20 Ma (Figure 3; Table 7).

The use of prior lognormal distributions for calibration of fossil ages plus soft maximum ages allows the relaxed clock method to correct for conflicting fossil-based time constraints. Particularly, the estimated range age of crown Platyrrhini, 21–30 to 27–34 Ma for the different hypotheses, differs significantly from the minimum fossil age of 12.5 Ma based on La Venta fauna. This result also differs significantly from a recent study that used similar fossil constraints [15], but with different lognormal distribution parameters and different maximum fossil constraints. Hodgson and co-workers [15] pointed out that “the lack of lower bounds (the maximum bound in the present work) within the platyrrhines fully allows for the data to support the MSH” (*morphological stasis hypothesis* or *long lineages hypothesis* of Rosenberger and co-workers [17]–[20]). However, the parameters of the prior distribution for calibrating fossil ages used by Hodgson and co-workers [15] generate a very narrow distribution that does not allow the support of the *long lineages hypothesis*. These results suggest that modeling the parameters of the prior lognormal distributions for calibration of fossil ages is very important in divergence time estimation. Therefore, our results support previous studies suggesting that a comprehensive divergence time estimation should account for uncertainty in – among other sources – fossil calibrations, parameters of the prior lognormal distribution and tree topology [21].

Particularly, the uncertainty in fossil calibrations should be carefully considered because it generates serious questions about the credibility of divergence time estimations [81]. Using fossils that are phylogenetically misplaced or that have incorrect ages can introduce serious error into molecular dating. Therefore, we need to use an explicit protocol to justify phylogenetic position and chronological age for fossil specimens [81]. Here, we provide a discussion about phylogenetic position and geochronological age of the most controversial platyrrhine fossils used as constraints in this and previous works [13]–[15], [25]. The above mentioned extinct *Dolichocebus* has indeed an age of 20 Ma, and in our view is linked to the cebids on the basis of cranial characters such as relatively narrow interorbital septum, relatively vaulted braincase, presence of an interorbital fenestra, as well as oval and vertically oriented orbits and a narrow face, and dental traits showing similarities to the Laventan *Neosamiri* and *Laventiana.* However, the natural status of some of these traits, such as the interorbital fenestra, is a matter of discussion [16]. In recent works, Kay and co-workers [16], [44], [70] argued that *Dolichocebus*, like all the other Patagonian platyrrhines except *Proteropithecia*, is part of the stem Platyrrhini. However, there is reason to believe their analysis and interpretation is negatively influenced by the difficulty of establishing legitimate anatomical similarities among specimens suffering from poor preservation of the edentulous type skulls of two crucial Patagonian taxa, *Dolichocebus* and *Tremacebus* [17].

The aotine status of *Tremacebus* is justified for us, especially for its relatively large orbits, strong postorbital constriction, and short and abbreviated face [28], [43]. However, Kay et al. [16], [82] suggest that the orbits are not significantly enlarged, as in *Aotus*, and they maintain that the olfactory bulb of *Tremacebus* (judging by the dimensions of its olfactory fossa) was also not enlarged as in *Aotus,* leading these authors to question the nocturnal status of *Tremacebus* and its phylogenetic link with *Aotus.* However, this view could be difficult to sustain, and considering that nocturnal habits are a secondary acquisition in *Aotus*, it is expected that this adaptation once presented a more primitive state, and *Tremacebus* has all the basic morphological patterns predicted to represent the ancestral pattern of the nocturnal adaptations. *Tremacebus* may have been not strictly nocturnal but cathemeral, like most species of the living *Aotus* [83], [84].

### Divergence Times Estimations Employing Body Size and Generation Time

By using generation times of extant New World monkey species, body size estimation for extant and extinct species and molecular rates directly observed in human families, we estimate rates of substitution per generation for the main platyrrhine lineages and the divergence times among these lineages without relying on external fossil calibration points. Our approach combines the Langergraber et al. [22] method which estimates divergence time from the estimated molecular rate per generation based on extant species, with the method proposed by Steiper and Seiffert [27] that corrects molecular rates in nuclear genomes of extant species using life-history variables, like body mass, inferred from the fossil record. We confirm the Steiper and Seiffert [27] and Langergraber et al. [22] observations about a relationship between body mass and generation times in primates using only platyrrhine data for extant species (*r* = 0.89). This corroborates the idea that body mass is correlated with generation time, and then substitution rate [27], assuming that most germ-line mutations occur during DNA replication.

Steiper and Seiffert [27] suggest that molecular rates slowed down over the course of primate evolution because they find an inverse relationship between body mass and molecular rate. However, our results show a different picture concerning the evolution of platyrrhine body size than the Steiper and Seiffert [27] obtained by averaging across all primates. Fossil platyrrhines display body size values similar to the living species, suggesting that body mass estimates of fossil platyrrhine species fall within the range expected for each of the extant main lineages. Therefore, the first platyrrhines of each lineage were approximately the size of the extant species of the same clade, supporting an ancient shift in body size for each clade, such as was shown recently in Aristide et al. [85]. Moreover, because of the strong correlation between body mass and generation time among platyrrhines, it is likely that generation times were approximately the same along the lineages evolution. Using this correlation we were able to predict the generation time of different platyrrhine lineages from ancestral reconstructions of body mass (Table 5), and then correct the estimation of molecular rates. In this sense our work does not assume that the generation times calculated for present-day primates are valid proxies for their ancestors, as in Langergraber et al. [22]. However, we assume that the molecular rates estimated from present-day human families can be used (plus body size and generation time) as a starting line to estimate and correct substitution rates among all extinct primates.

The divergence time estimates based on the molecular rate for Old World primates are also in agreement with those obtained in a recent report (Table 8; [22]), suggesting that the method is also useful for the analyzed Perelman et al. [26] dataset, a stratified sample of the nuclear genome. Similarly, divergence time estimates for the platyrrhine LCA show ranges that include the estimates based on the relaxed-clock model and fossil constraints (Table 8), being closer in age to the earliest undoubted fossil platyrrhine, *Branisella* (*ca.* 26 Ma), and to the direct interpretations of the fossil record [19], [28]. Particularly, we estimate that the LCA of extant platyrrhine monkeys existed between *ca.* 21–29 Ma. Additionally, we estimate the branching of Atelidae between *ca.* 19–27 Ma and of the Cebidae LCA between *ca.* 16–22 Ma (Table 8). These results suggest that the previous differences observed in the length of platyrrhine branches [15], [60] compared with catarrhines is related to differences in generation time and not to time of divergence. It is important to highlight that by using a very wide range of molecular rates for extinct platyrrhine taxa – estimations based on conservative values of body size and generation time – we generated a wide range of divergence time uncertainty, similar to the BEAST estimations (see [22]).

**Table 8:** Generation based divergence time estimations.

Node	Lower and higher substitution rate	Lower and higher divergence time estimation
Crown Platyrrhini	8.5E-010–6.06E-010	20.31–28.49
Atelidae branching	8.5E-010–6.06E-010	19.05–26.72
Crown Cebidae	9.07E-010–6.47E-010	15.56–21.81
Crown Anthropoidea	8.5E-010–6.06E-010	36.88–51.73
Crown Catarrhini	6.97E-010–4.97E-010	24.29–34.06
*Homo/Pan*	5.04E-10–3.59E-010	7.31–10.26

Intervals of divergence times (in millions of years) for selected nodes in the platyrrhine tree under alternative substitution rate.

### Divergence Time and the Platyrrhine Radiation

As pointed out in the Introduction section, dating the basal crown platyrrhine has implications for understanding the platyrrhine evolutionary radiation. Our results suggest that molecular divergence times generated using fossil constraints and molecular rate information are not enough to confidently reject the hypothesis that crown Platyrrhini and the main platyrrhine lineages could have diverged at or before 20 Ma. This result is in marked contrast with the results of Hogdson et al. [15] molecular study. Therefore, molecular divergence time estimations cannot be used to support the idea that platyrrhine diversification is characterized by two successive, sister-group radiations [16] and to contradict the *long lineages hypothesis* of Rosenberger and co-workers [17]–[20].

In a recent submitted work [85] we explore other dimensions of platyrrhine diversification, such as the tempo and mode of species origination and the dynamics of body size evolution. In it, evidence is presented that suggests that platyrrhine evolution conforms to an adaptive radiation model, in which lineages are accumulated at a high rate during the early stages of a clade’s evolutionary history. Moreover, body size variation is shown to have been partitioned among subclades early in the phylogenetic history of the platyrrhines, a pattern that is also in agreement with an adaptive radiation scenario and with body size estimations for fossil specimens. Taken together, the results of Aristide et al. [85] and the results of the present work, where we show that extant lineages probably have an ancient origin, are complementary to extend our understanding of the platyrrhine diversification history and stress the role of morphological stasis as a deep evolutionary phenomenon, providing new evidence that contribute to the long standing debate between contrasting hypotheses (*long lineages* vs. *successive radiations*).

### Conclusion

In this work we used two largely independent molecular approaches (calibration bounds using BEAST and external molecular rates) to estimate the initial divergence time of platyrrhines. Both approaches have advantages and questions [86]. The approach based on calibration bounds using BEAST has the advantage of being relatively sequence independent, but indisputable and reliable calibration bounds are rarely available [21], [86]. The approach based on external molecular rates has the advantage of not requiring such calibration bounds [22], [27], [86]. However, dating methods based on external molecular rate estimations are in their initial stages of development and therefore not free of questions; particularly because they would yield younger divergence estimates than given for other methods (see [87], [88]). Nevertheless, our findings show that these methods are promising.

Our results suggest that several interpretations of the relationships between extant species and the ancient Patagonian fossil record are probably correct [17], [19], [20], [28], [58]. We also conclude that although the current platyrrhine fossil record is relatively scarce, it is not necessarily poorly sampled [7], [28], [89].The estimations based on the two approaches used in this study recalibrate the ages of the platyrrhine clades and make it possible to reconcile several points concerning the affinities of key fossils that have been contested. Contrary to the work of Hodgson et al. [15], our present work includes *Branisella boliviana* (*ca.* 26 Ma), which may fall within the platyrrhine crown group; *Dolichocebus gaimanensis* (*ca.* 20 Ma), which may represent the cebine lineage; and *Tremacebus harringtoni* (*ca.* 20 Ma), which may be an aotine. However, these estimates cannot resolve the controversy of whether these fossil species truly belong to the extant lineages or to closely related lineages [22], [81]. While that question can only be resolved by morphology, our study provides additional evidence that makes likely the broader evolutionary hypothesis that platyrrhine differentiation unfolded as a series of long-lived lineages with morphological stasis [19]. More generally, we show that the use of different approaches, considering molecular rate, fossil record and generation time, gives a more robust divergence time estimation for a clade and allows a more detailed discussion of its biological diversification.

## Supporting Information

Figure S1 **Wildman-BEAST chronophylogenetic trees.** Chronophylogenetic trees from the BEAST analysis for 28 species of Primates based on mtDNA and nuclear sequences and using monophyly constraints based on Wildman et al. [69] and alternative fossil calibrations (see table 4). Mean node ages are depicted in each node. Blue horizontal bars represent the posterior 95% CI for the node ages. The vertical line shows the estimated earliest age of Patagonian lineages.
(PDF)

Figure S2 **Perelman-BEAST chronophylogenetic trees.** Chronophylogenetic trees from the BEAST analysis for 28 species of Primates based on mtDNA and nuclear sequences and using monophyly constraints based on Perelman et al. [26] and alternative fossil calibrations (see table 4). Mean node ages are depicted in each node. Blue horizontal bars represent the posterior 95% CI for the node ages. The vertical line shows the estimated earliest age of Patagonian lineages.
(PDF)

Figure S3 **Opazo-BEAST chronophylogenetic trees.** Chronophylogenetic trees from the BEAST analysis for 28 species of Primates based on mtDNA and nuclear sequences and using monophyly constraints based on Opazo et al. [13] and alternative fossil calibrations (see table 4). Mean node ages are depicted in each node. Blue horizontal bars represent the posterior 95% CI for the node ages. The vertical line shows the estimated earliest age of Patagonian lineages.
(PDF)

Figure S4 **Rosenberger-BEAST chronophylogenetic trees.** Chronophylogenetic trees from the BEAST analysis for 28 species of Primates based on mtDNA and nuclear sequences and using monophyly constraints based on Rosenberger [10], [18] and alternative fossil calibrations (see table 4). Mean node ages are depicted in each node. Blue horizontal bars represent the posterior 95% CI for the node ages. The vertical line shows the estimated earliest age of Patagonian lineages.
(PDF)

Figure S5 **Quadratic curve.** Plot showing body mass and generation time for extant species (red symbols), and body mass and imputed generation time for fossil taxa (blue symbols) using a quadratic curve.
(PDF)

Figure S6 **Linear regression fit.** Plot showing body mass and generation time for extant species (red symbols), and body mass and imputed generation time for fossil taxa (blue symbols) using a linear regression (OLS) fit.
(PDF)

Figure S7 **EM fit.** Plot showing body mass and generation time for extant species (red symbols), and body mass and imputed generation time for fossil taxa (blue symbols) using a EM fit.
(PDF)

Table S1 **OLS results.** OLS Regression results for extant taxa.
(PDF)

Material S1 **Regression between body mass and generation time, imputation procedure and molecular rate correction.**
(PDF)
