# Supplementary figures and images for: Divergence Times and the Evolutionary Radiation of New World Monkeys (Platyrrhini, Primates): An Analysis of Fossil and Molecular Data
Source: PLoS One. 2013 Jun 27;8(6):e68029. doi: 10.1371/journal.pone.0068029 (PMC3694915; doi:10.1371/journal.pone.0068029)

Nuclear

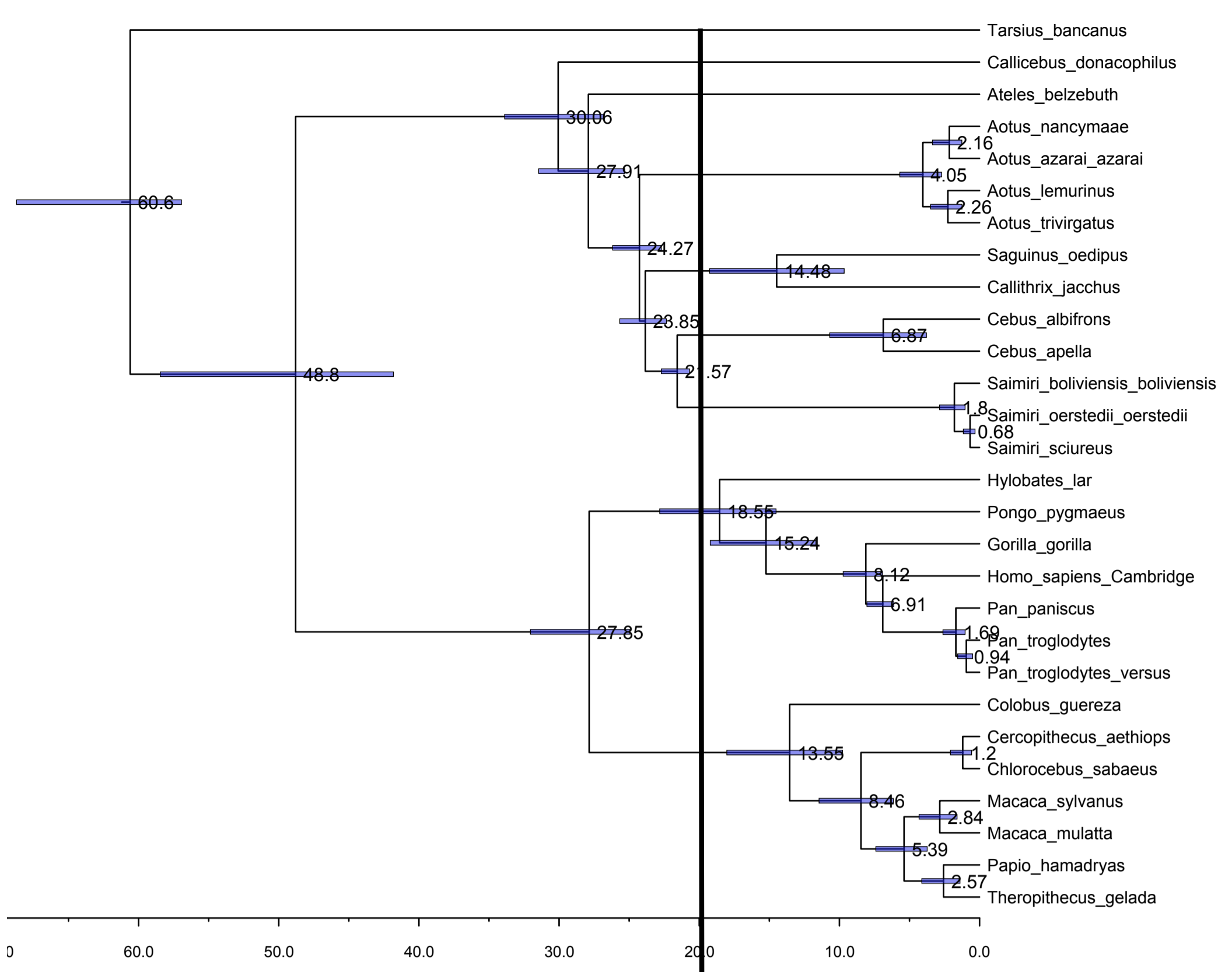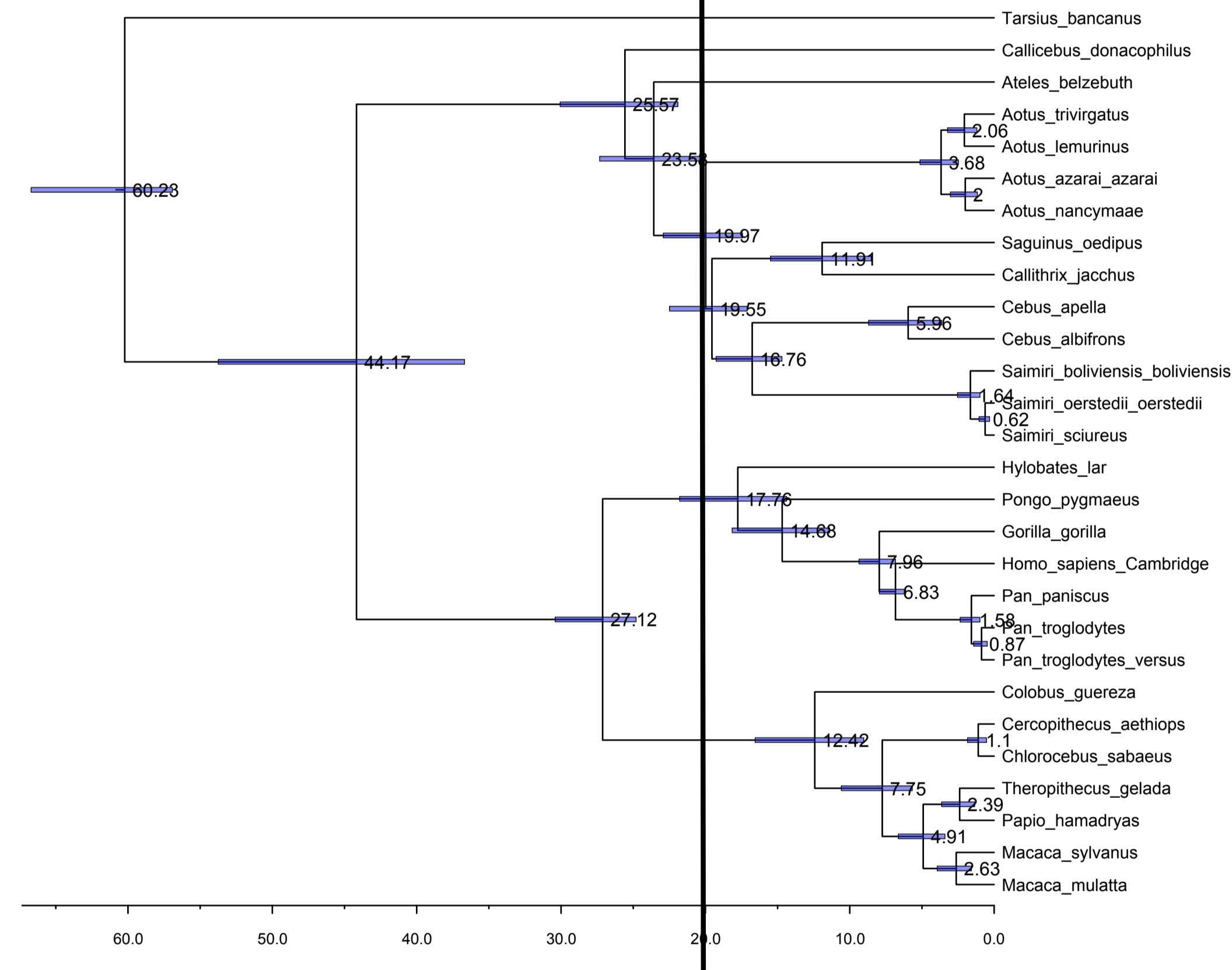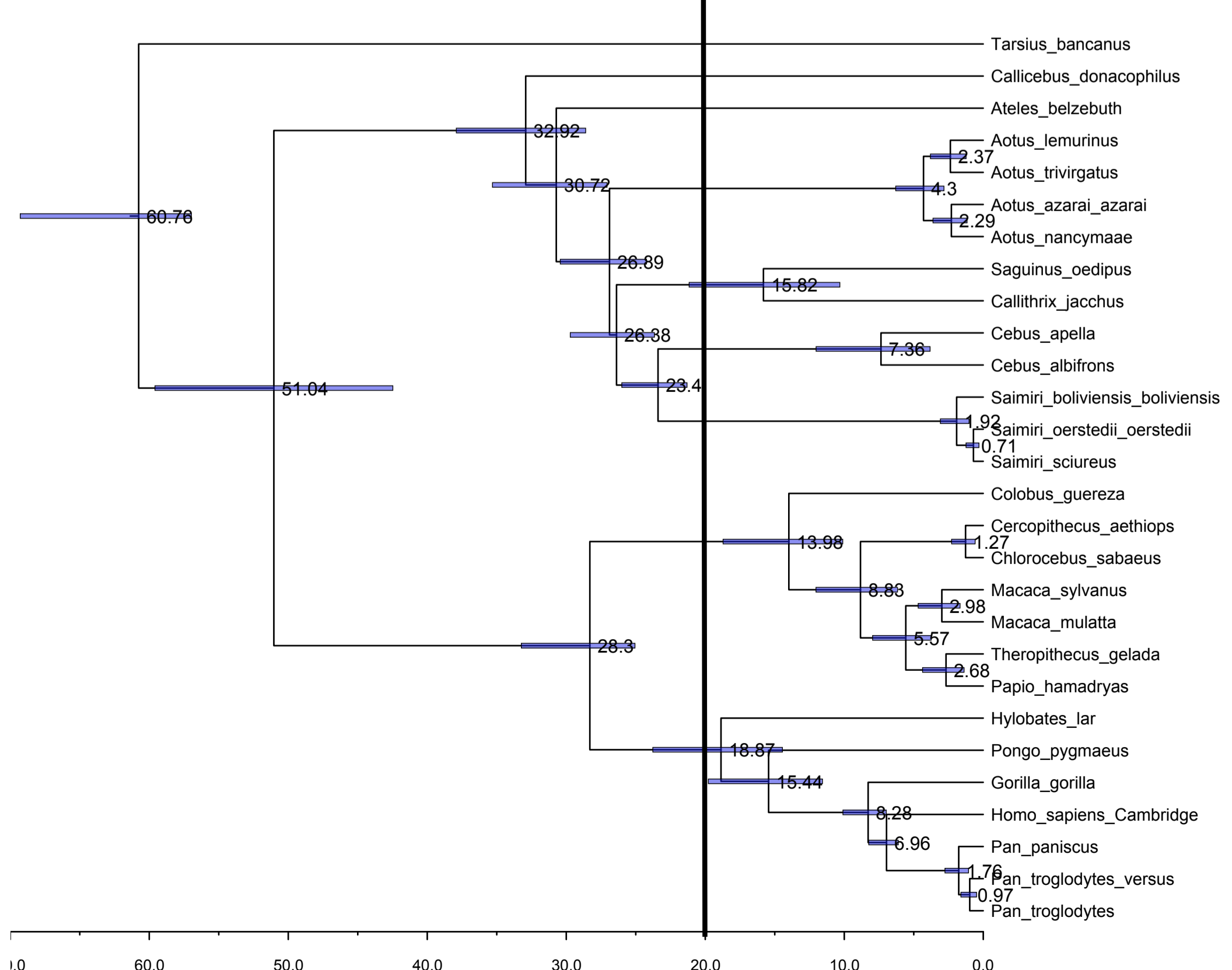

mtDNA

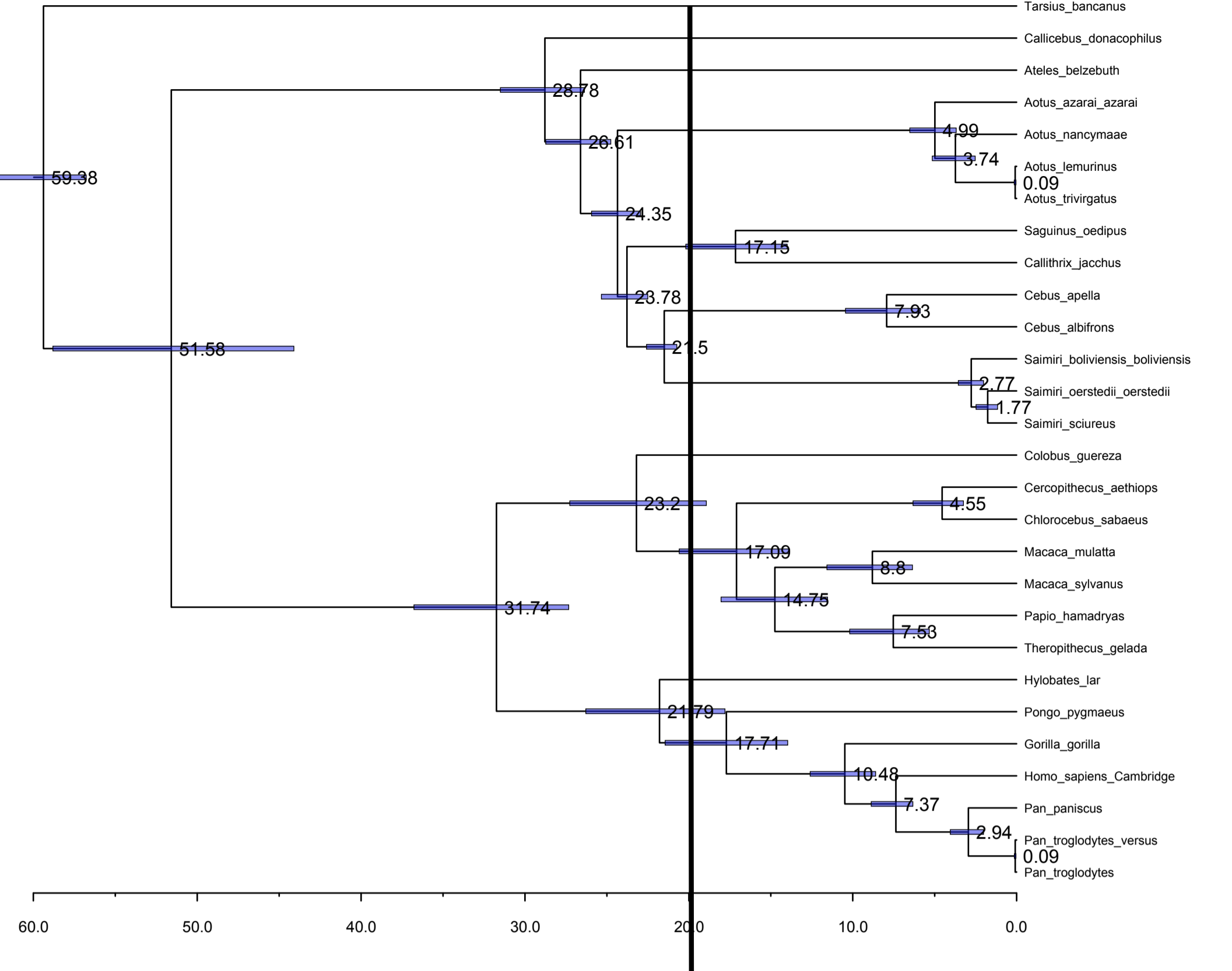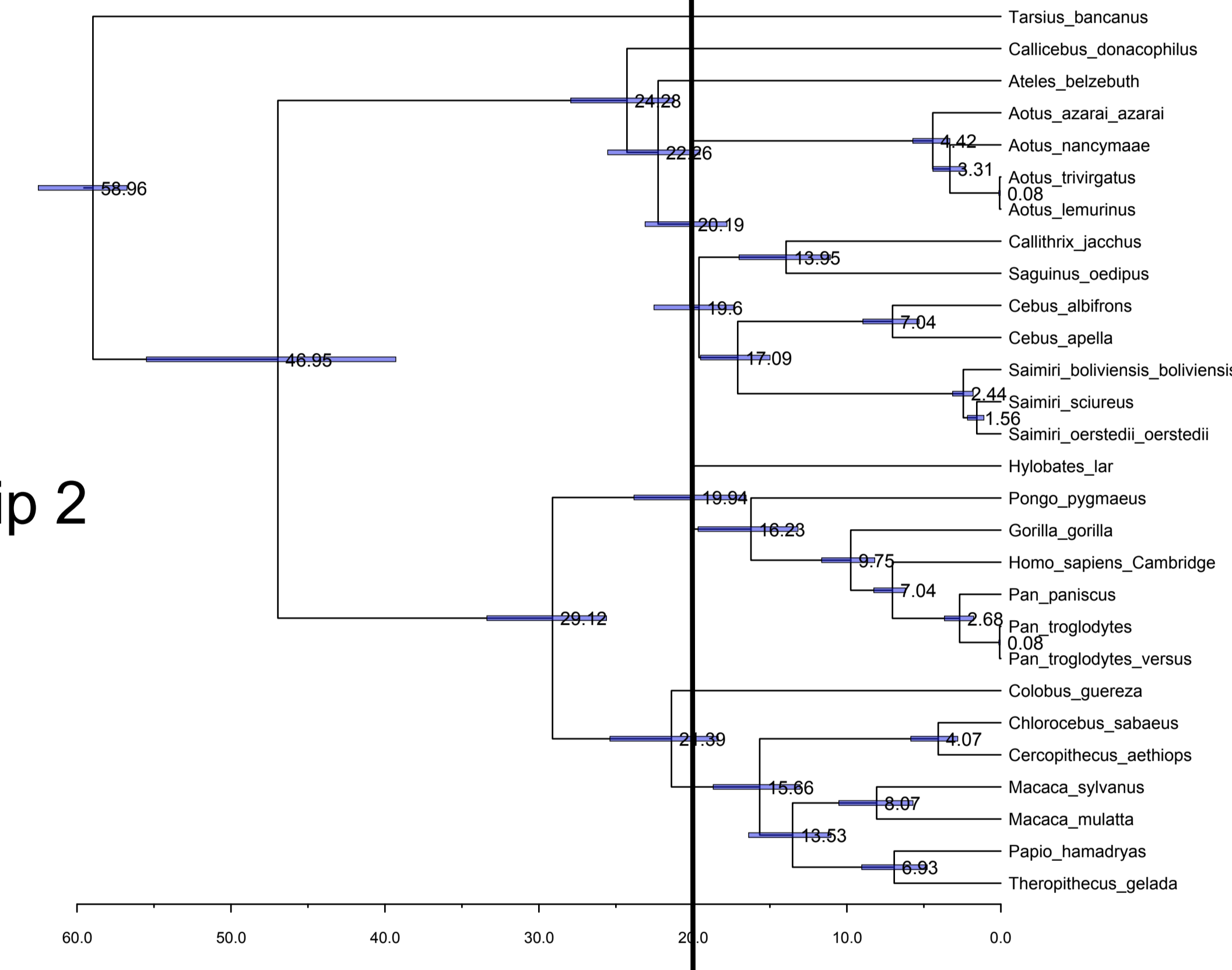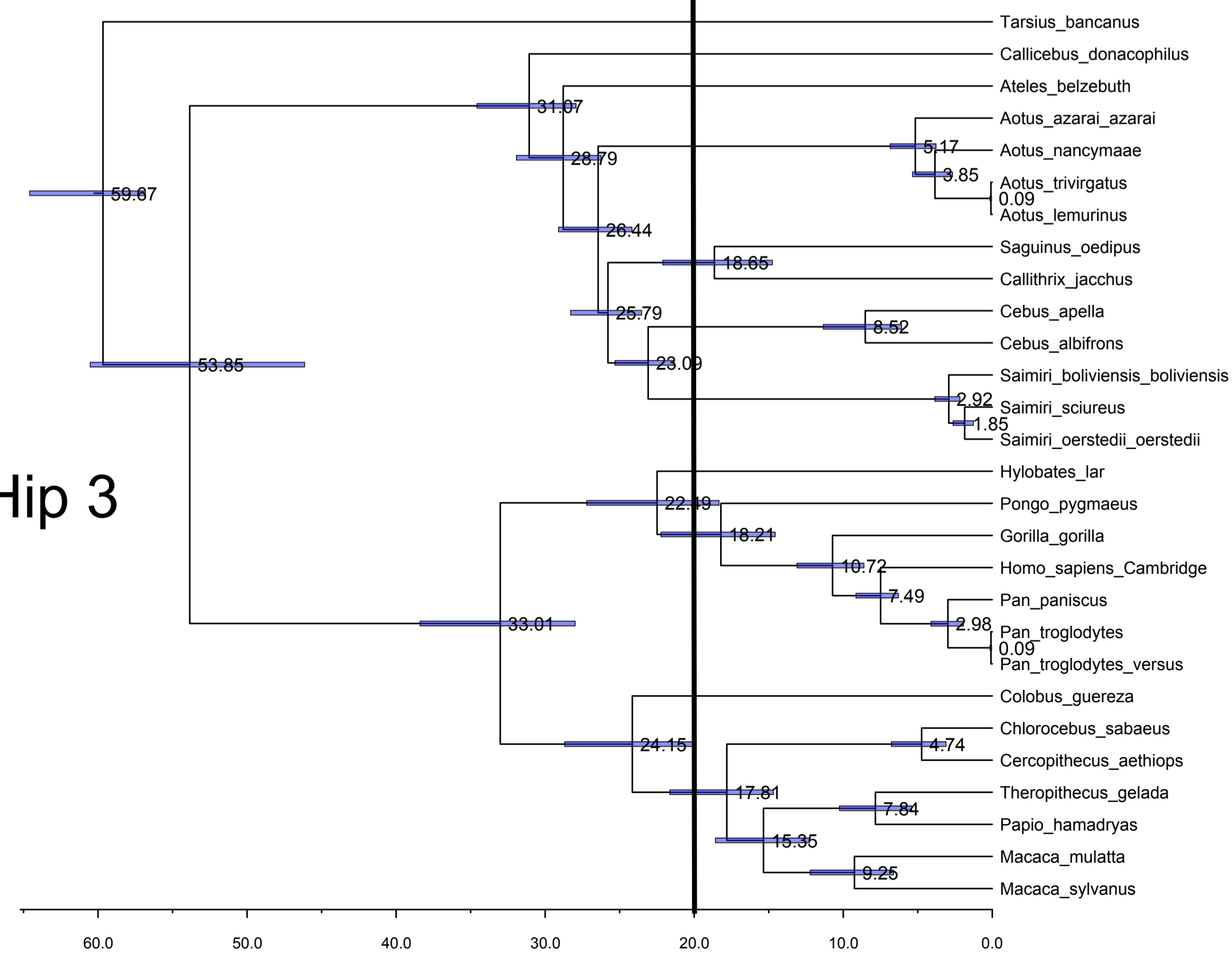

Hip 1

Hip 2

Hip 3

Supplement: Figure S1 — Wildman-BEAST chronophylogenetic trees. Chronophylogenetic trees from the BEAST analysis for 28 species of Primates based on mtDNA and nuclear sequences and using monophyly constraints based on Wildman et al. [69] and alternative fossil calibrations (see table 4). Mean node ages are depicted in each node. Blue horizontal bars represent the posterior 95% CI for the node ages. The vertical line shows the estimated earliest age of Patagonian lineages. (PDF) [file pone.0068029.s001.pdf]

Nuclear

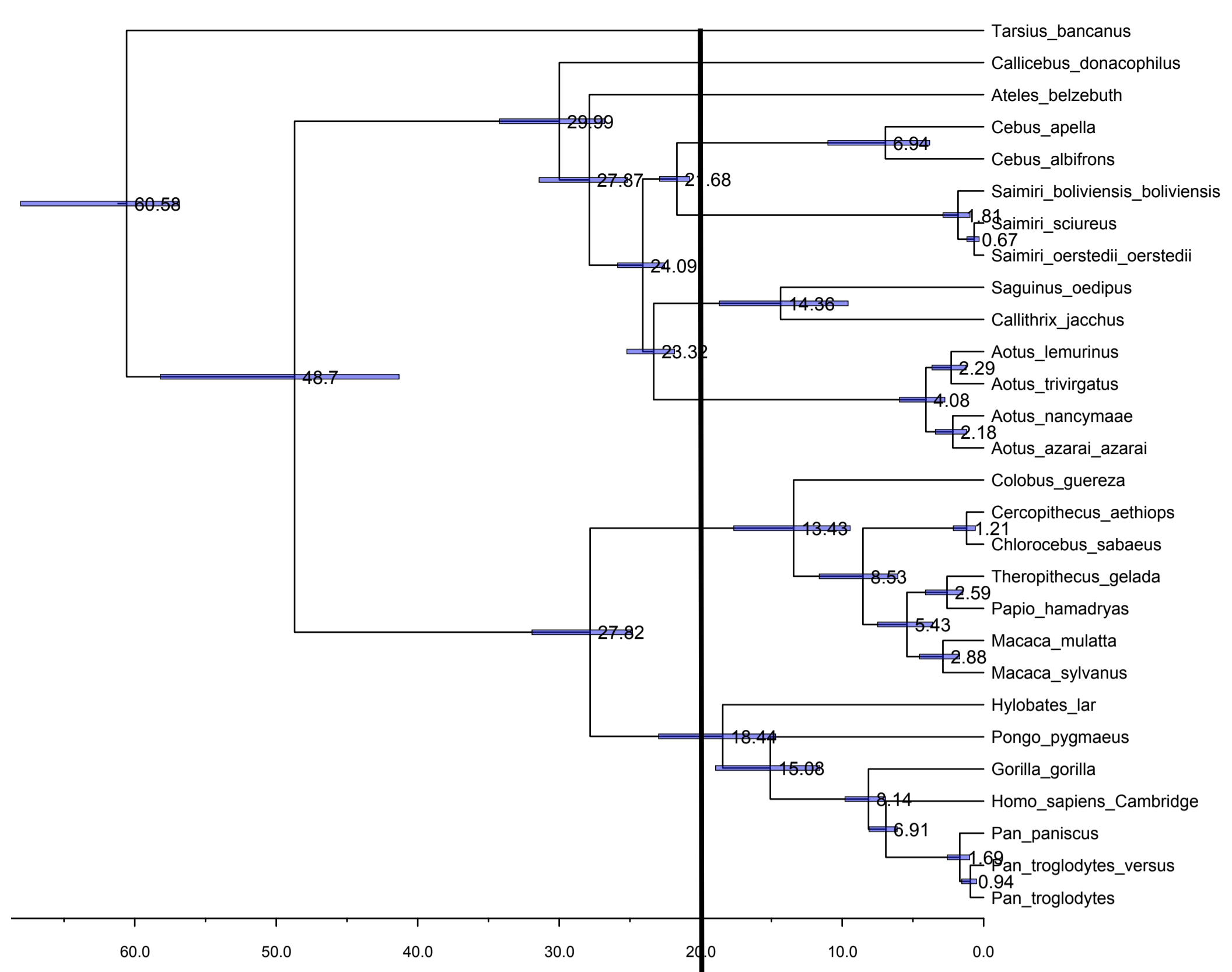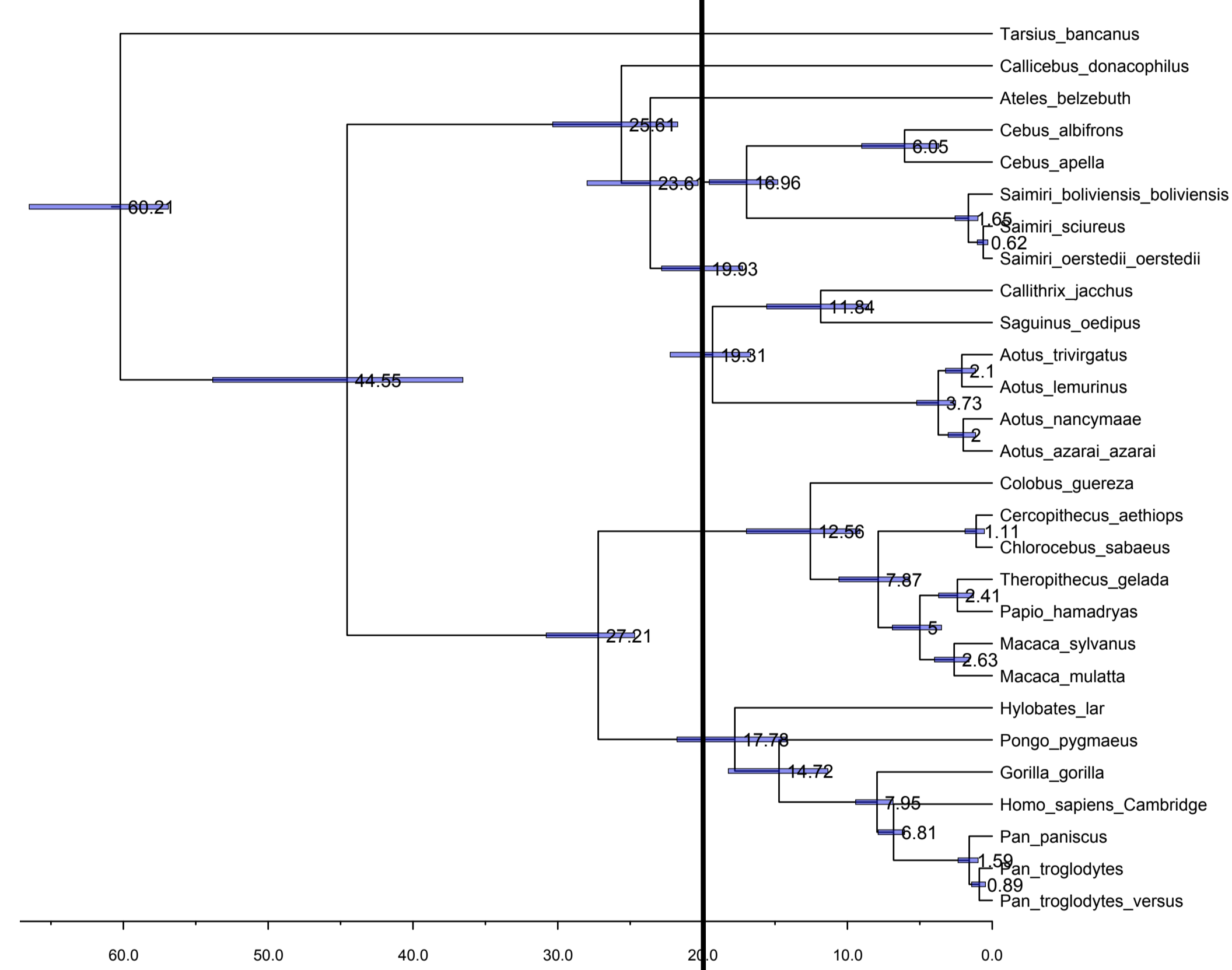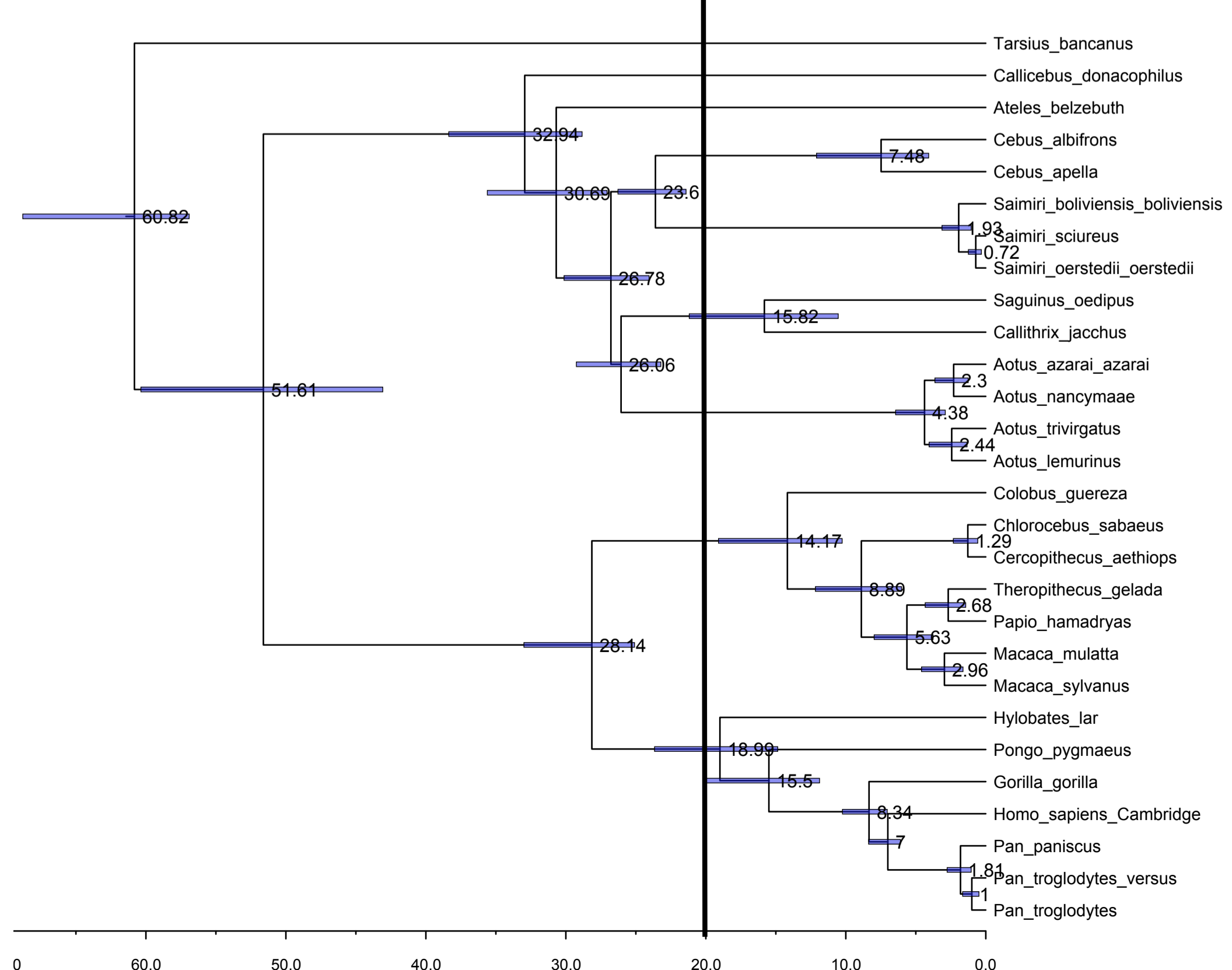

mtDNA

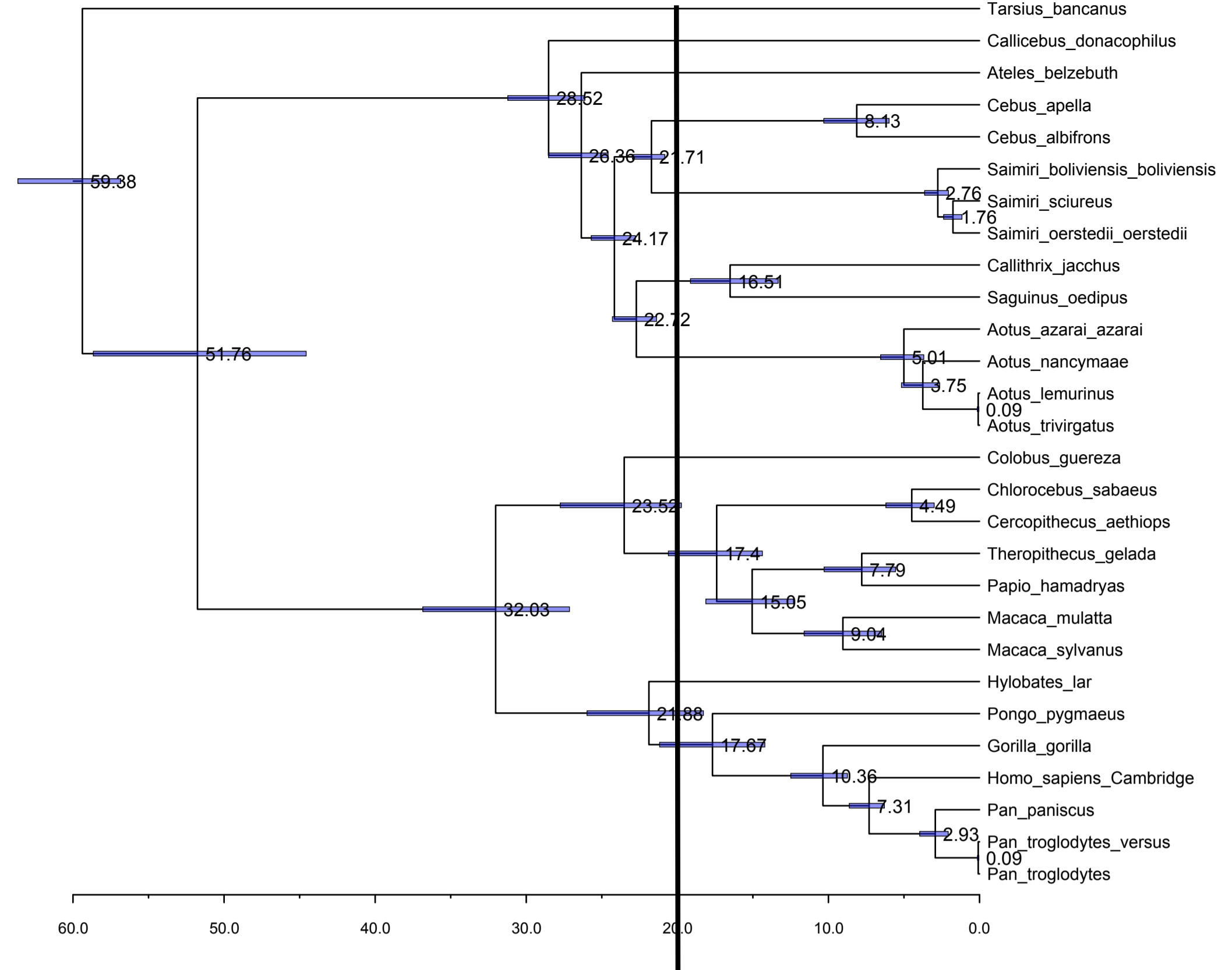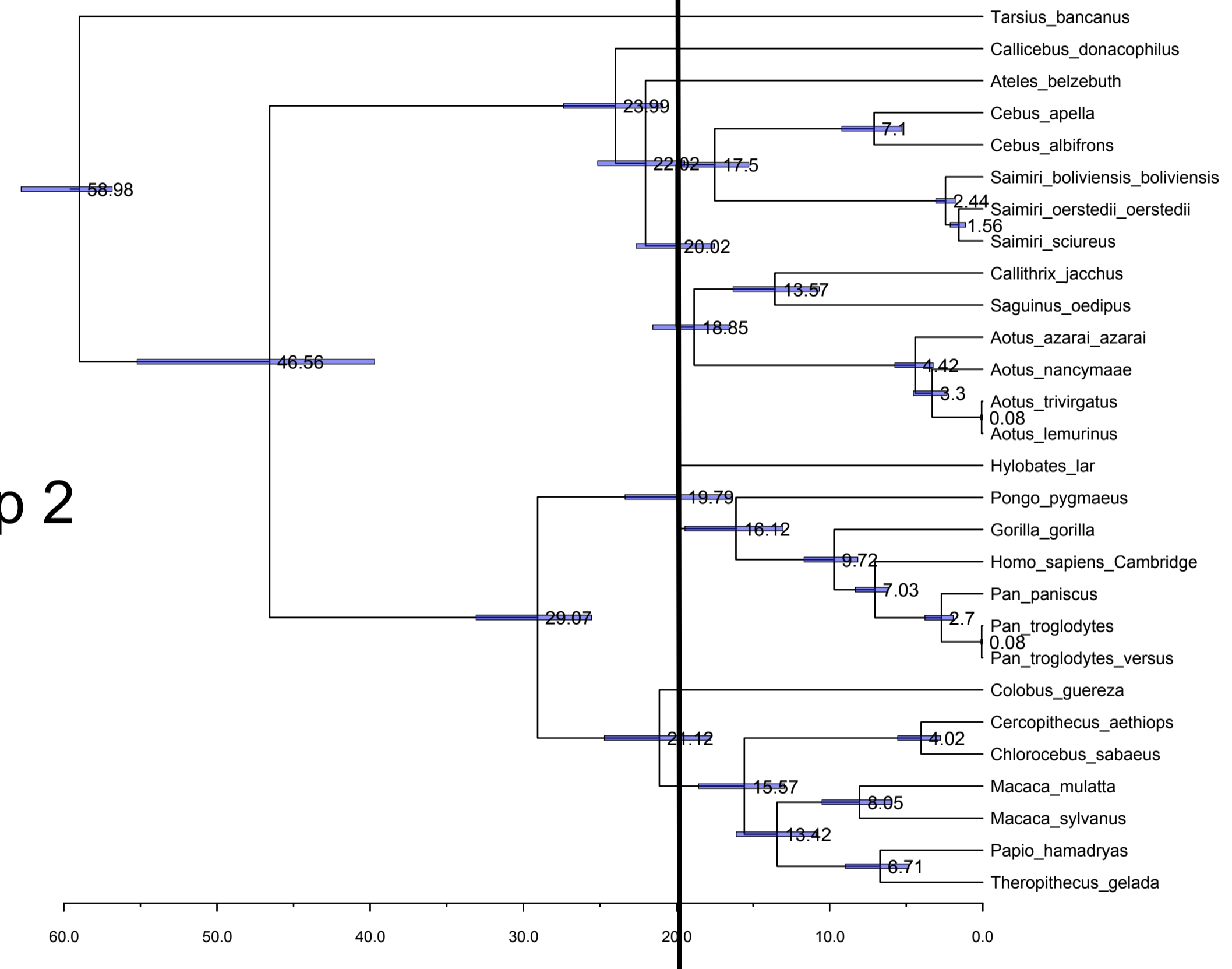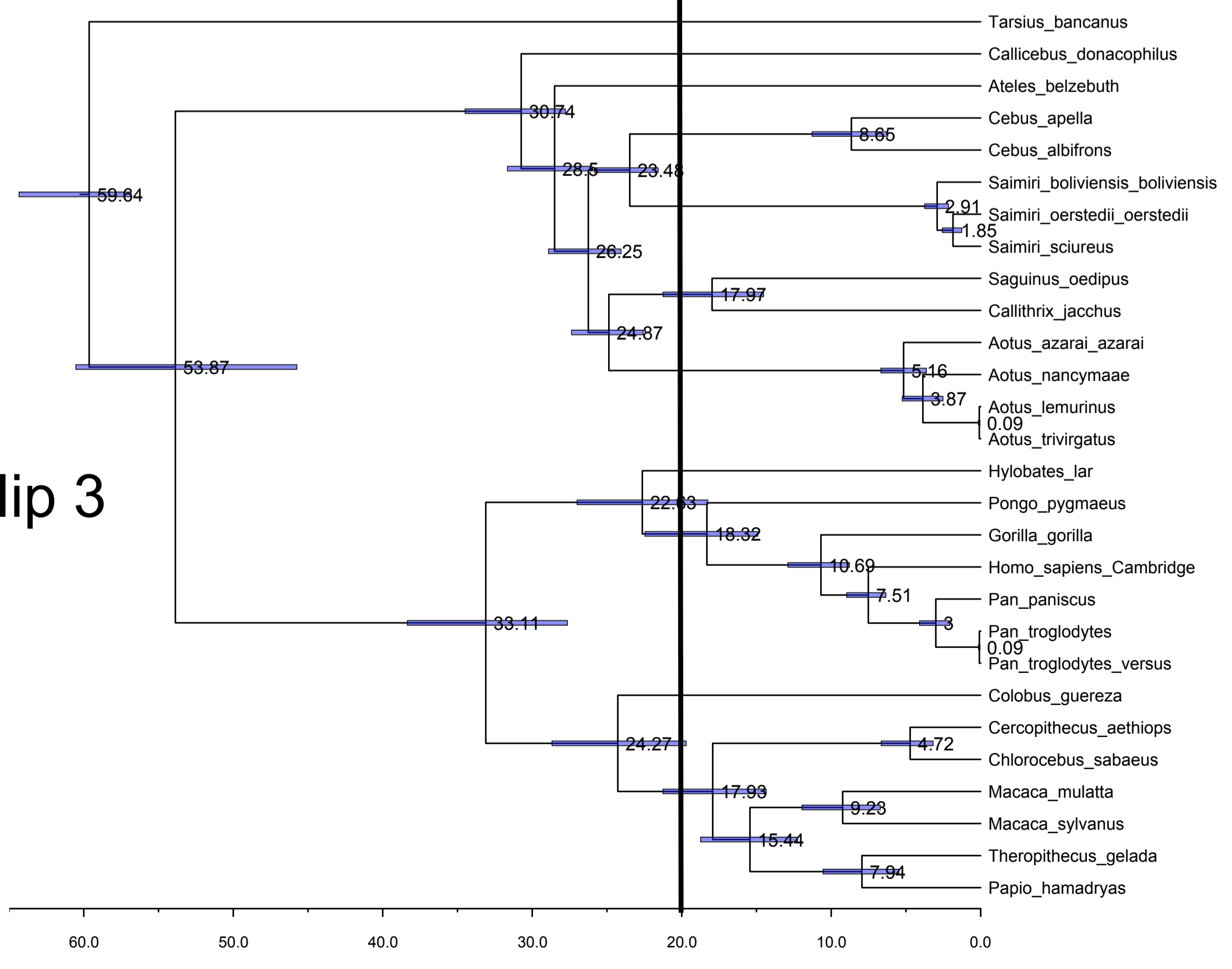

Hip 1

Hip 2

Hip 3

Supplement: Figure S2 — Perelman-BEAST chronophylogenetic trees. Chronophylogenetic trees from the BEAST analysis for 28 species of Primates based on mtDNA and nuclear sequences and using monophyly constraints based on Perelman et al. [26] and alternative fossil calibrations (see table 4). Mean node ages are depicted in each node. Blue horizontal bars represent the posterior 95% CI for the node ages. The vertical line shows the estimated earliest age of Patagonian lineages. (PDF) [file pone.0068029.s002.pdf]

Nuclear

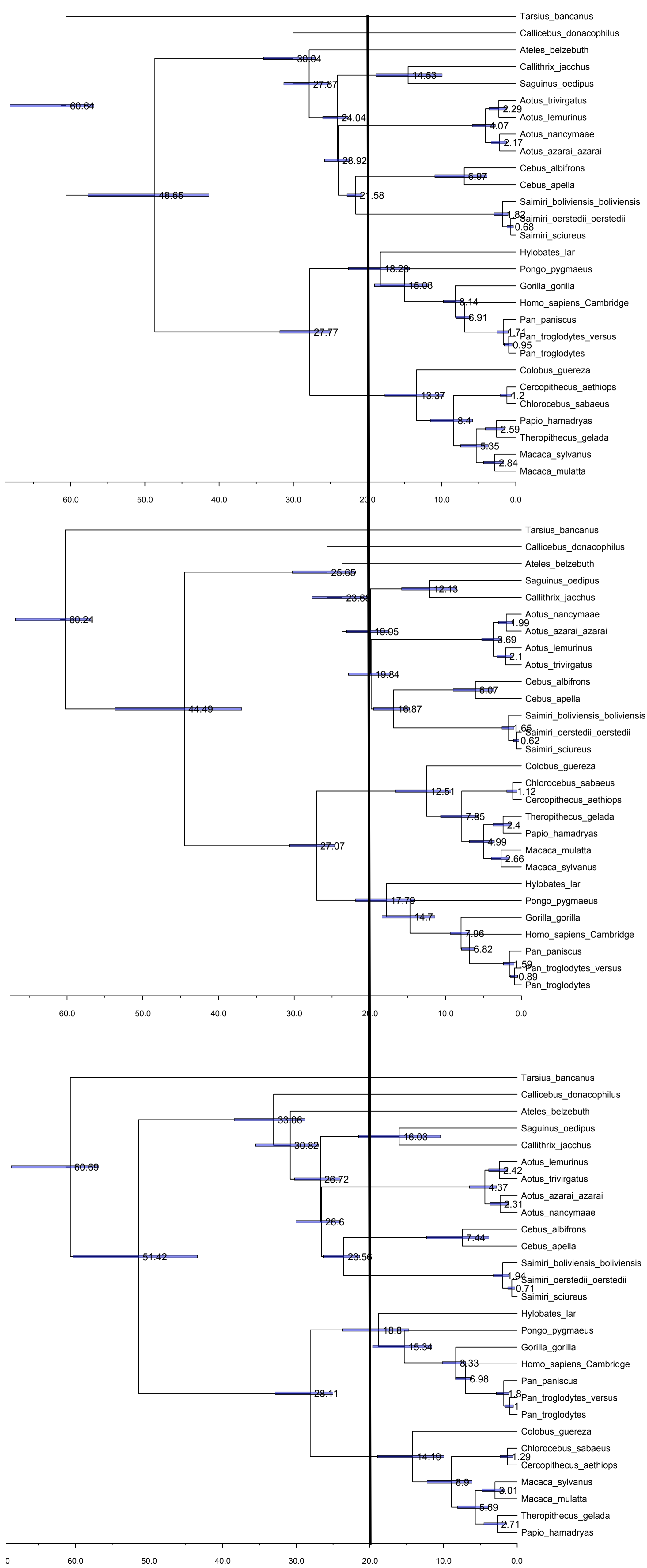

mtDNA

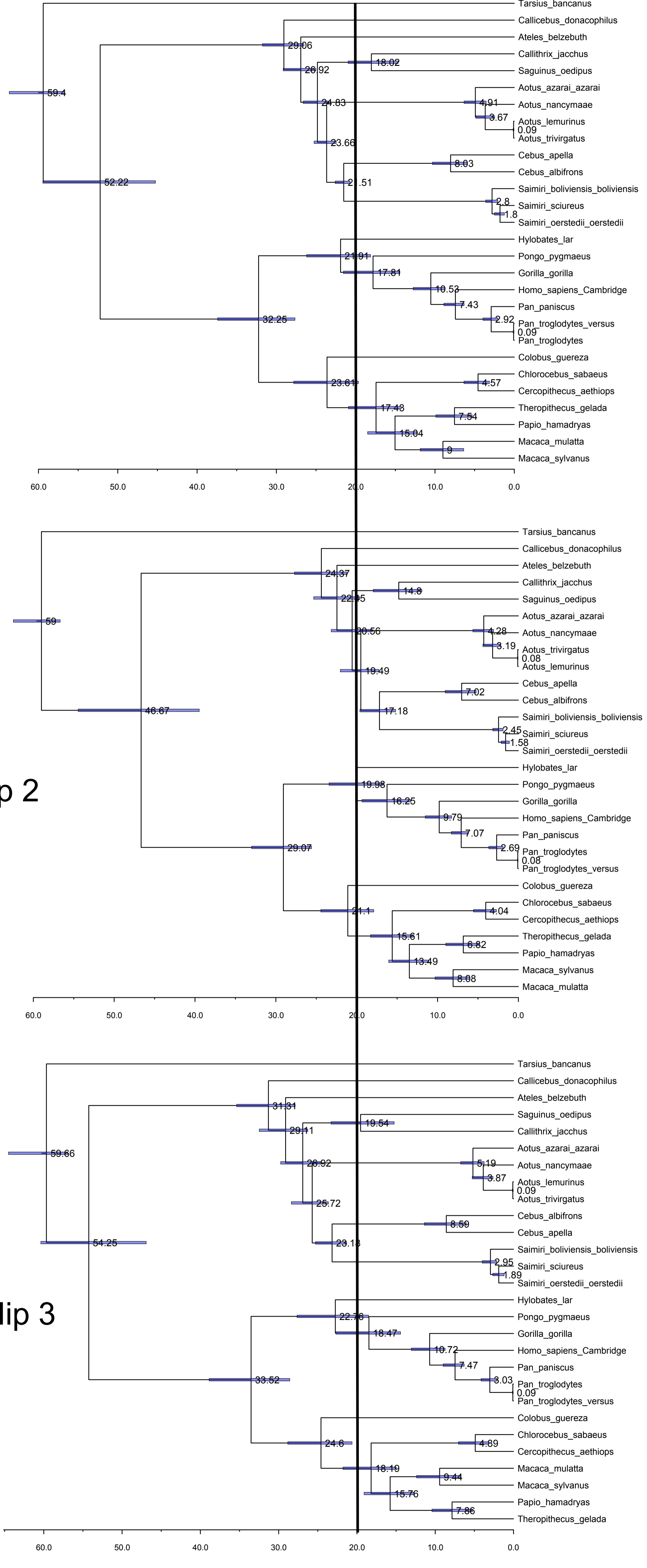

Hip 1

Hip 2

Hip 3

Supplement: Figure S3 — Opazo-BEAST chronophylogenetic trees. Chronophylogenetic trees from the BEAST analysis for 28 species of Primates based on mtDNA and nuclear sequences and using monophyly constraints based on Opazo et al. [13] and alternative fossil calibrations (see table 4). Mean node ages are depicted in each node. Blue horizontal bars represent the posterior 95% CI for the node ages. The vertical line shows the estimated earliest age of Patagonian lineages. (PDF) [file pone.0068029.s003.pdf]

Nuclear

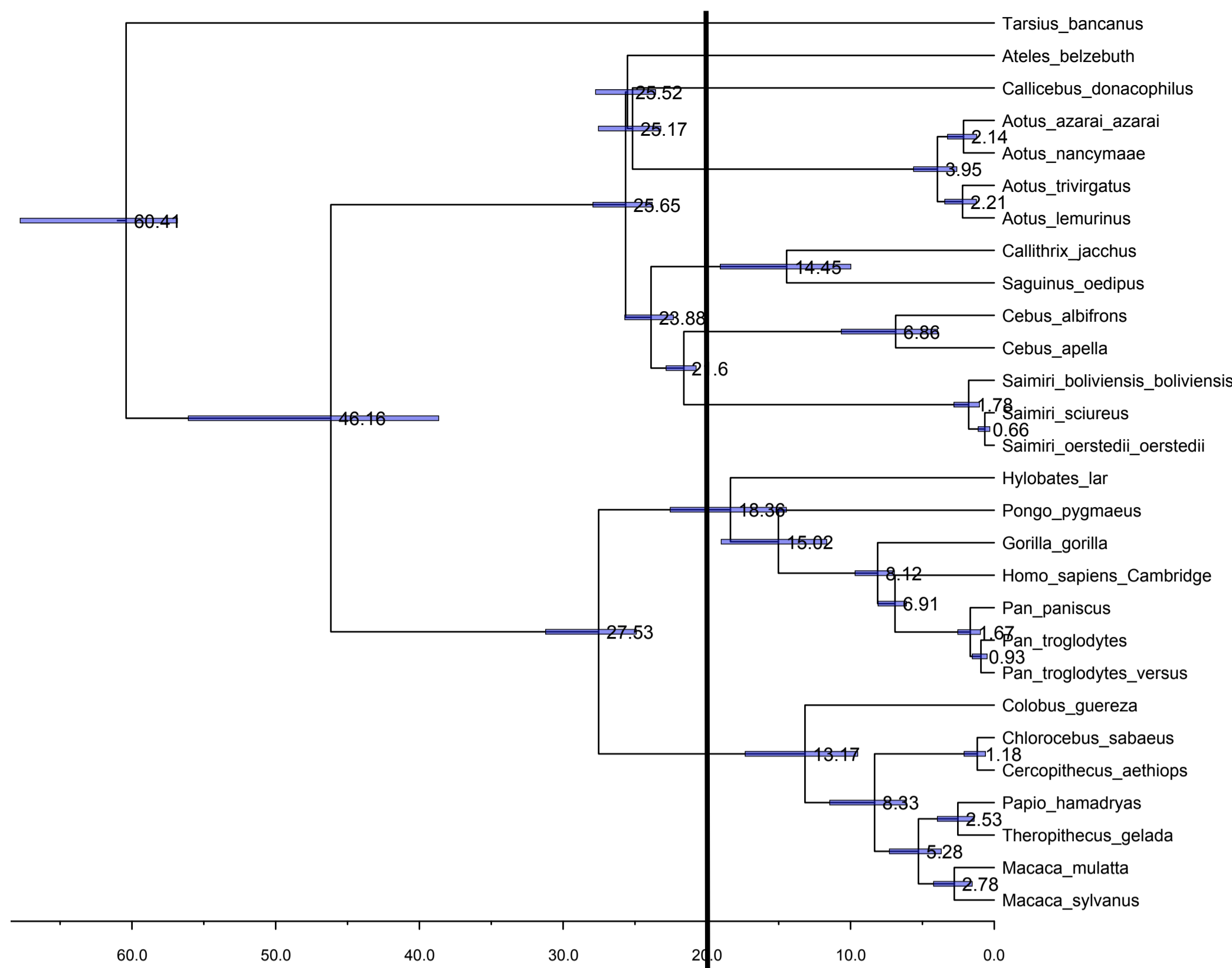

mtDNA

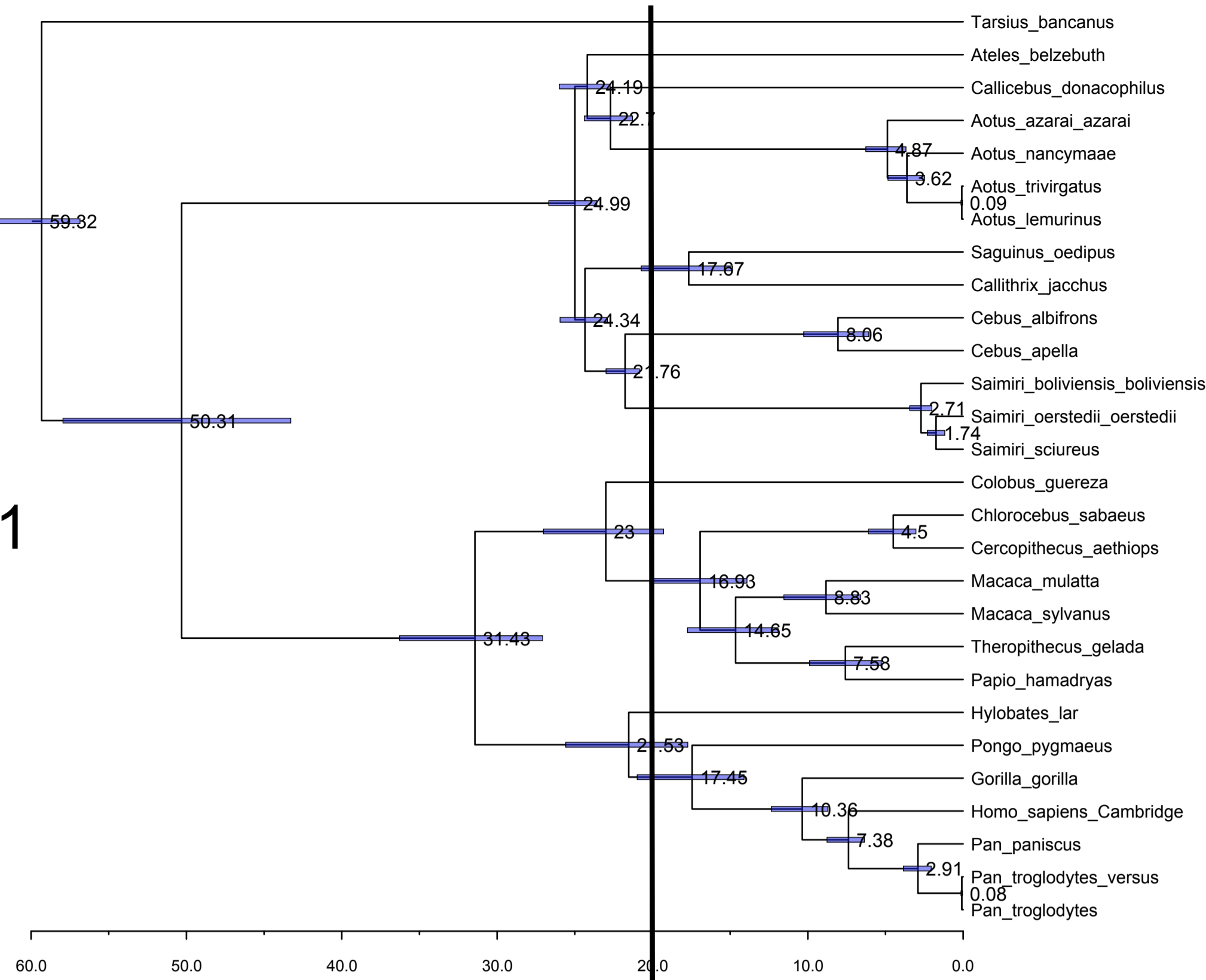

Hip 1

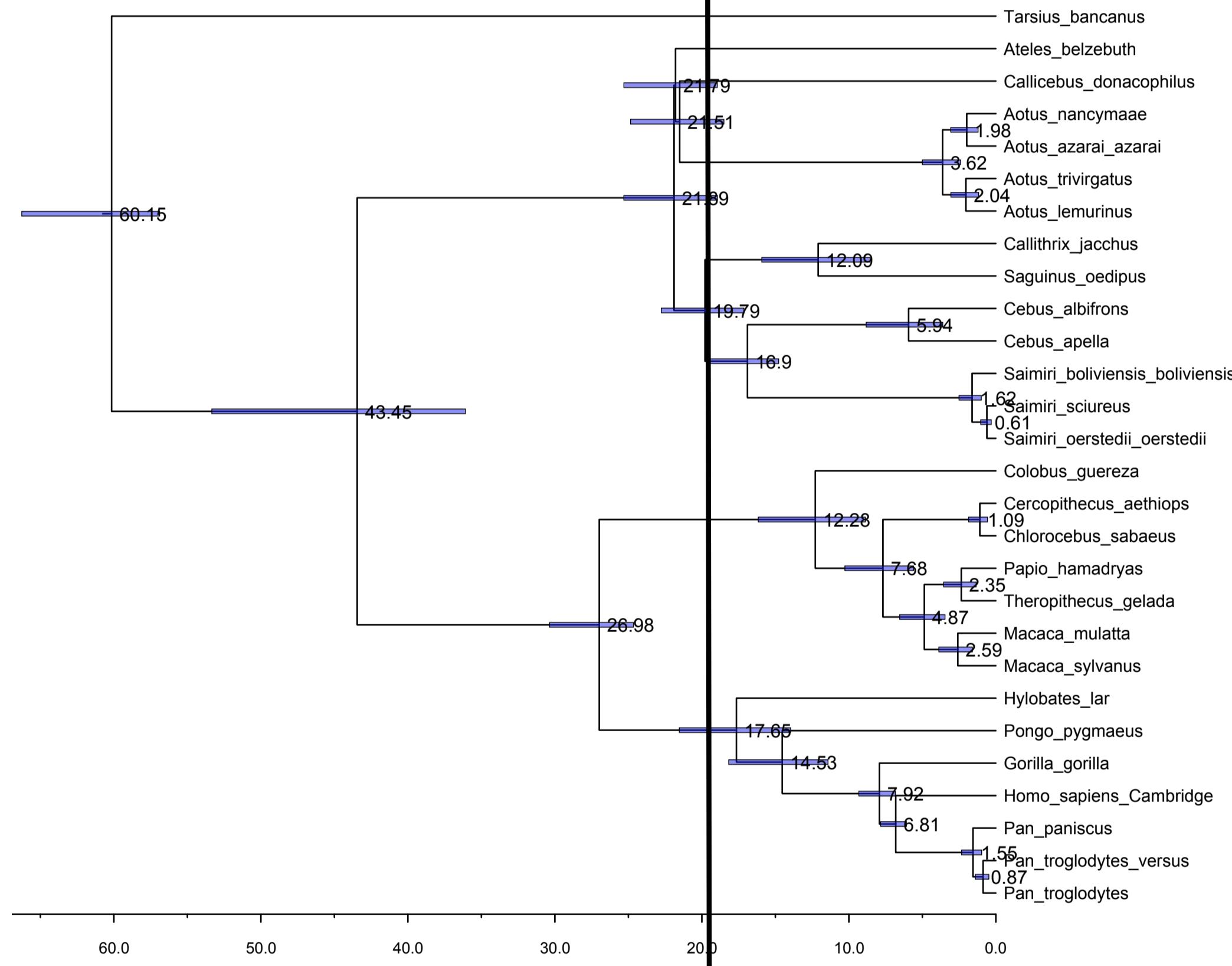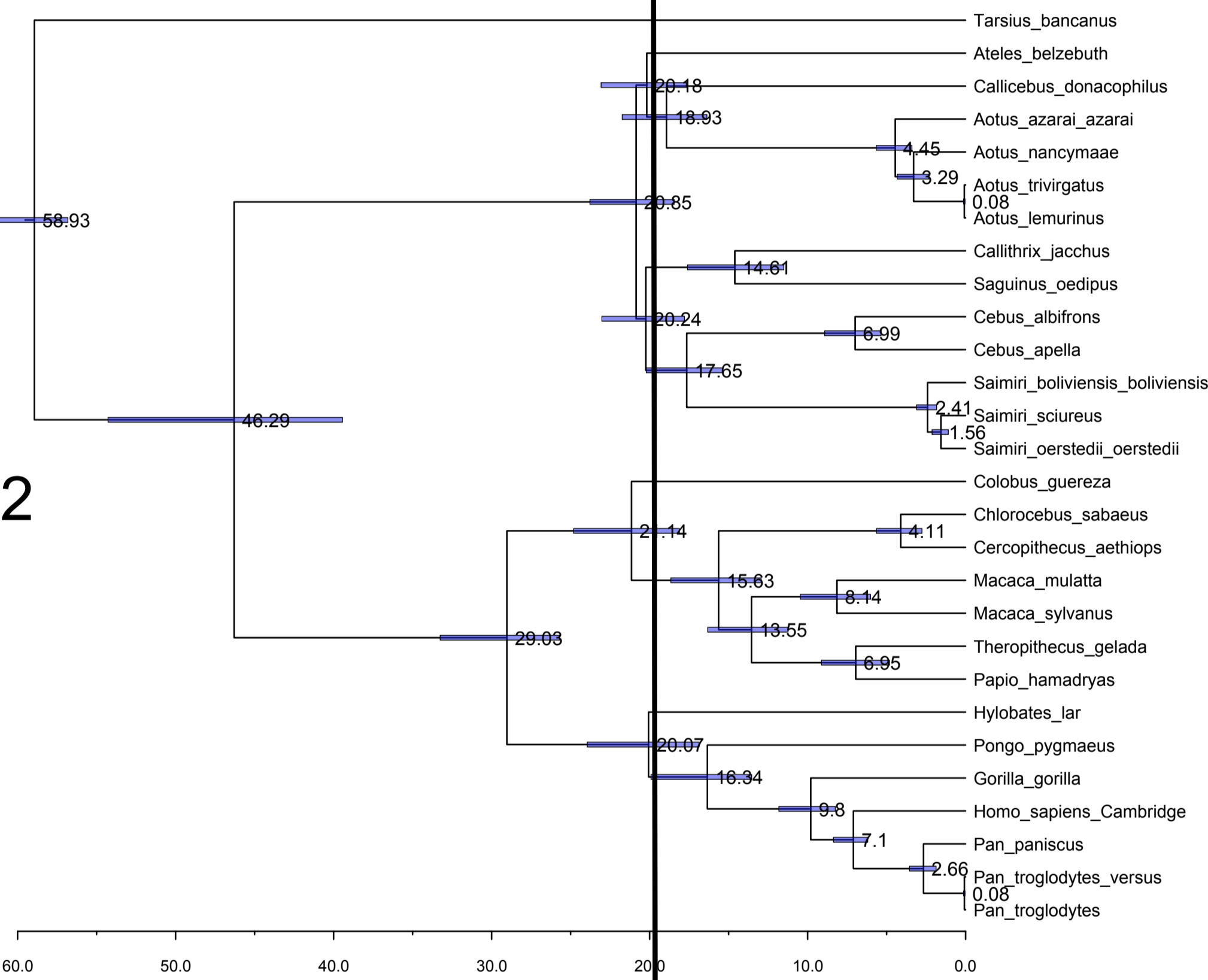

Hip 2

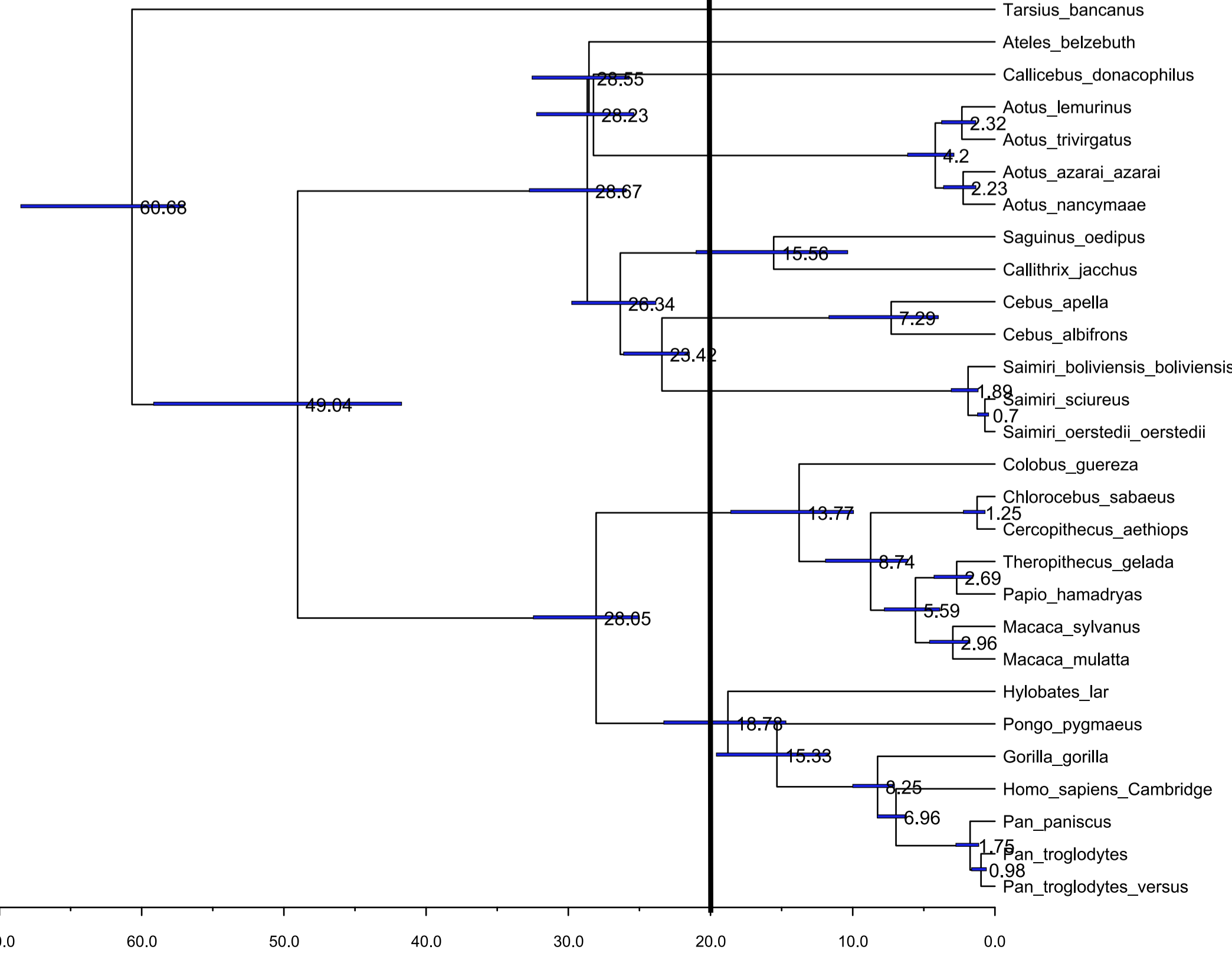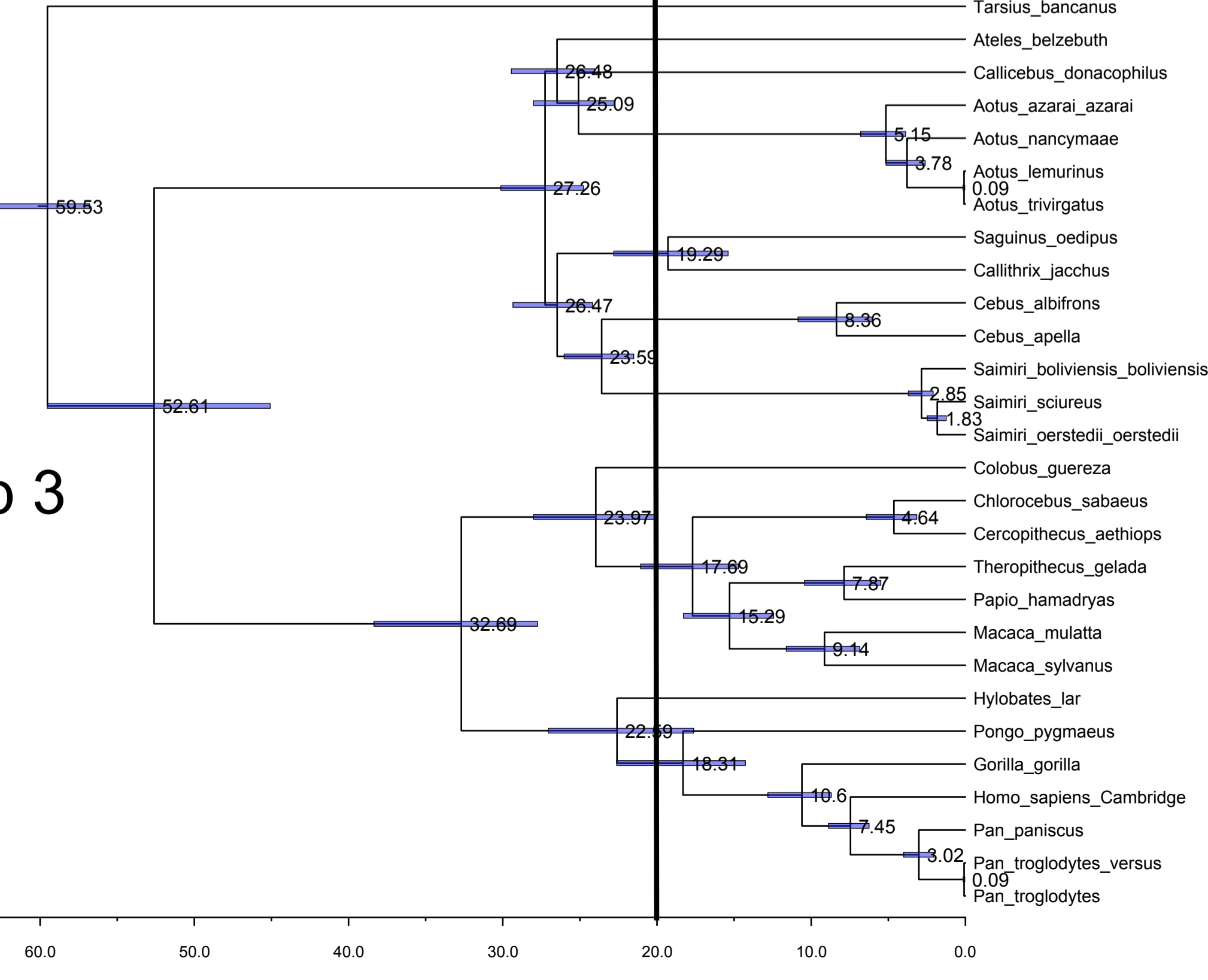

Hip 3

Patagonian Fossils

Patagonian Fossils

Supplement: Figure S4 — Rosenberger-BEAST chronophylogenetic trees. Chronophylogenetic trees from the BEAST analysis for 28 species of Primates based on mtDNA and nuclear sequences and using monophyly constraints based on Rosenberger [10], [18] and alternative fossil calibrations (see table 4). Mean node ages are depicted in each node. Blue horizontal bars represent the posterior 95% CI for the node ages. The vertical line shows the estimated earliest age of Patagonian lineages. (PDF) [file pone.0068029.s004.pdf]
