# Supplementary material for: Divergence Times and the Evolutionary Radiation of New World Monkeys (Platyrrhini, Primates): An Analysis of Fossil and Molecular Data
Source: PLoS One. 2013 Jun 27;8(6):e68029. doi: 10.1371/journal.pone.0068029 (PMC3694915; doi:10.1371/journal.pone.0068029)
Supplement: Figure S6 — Linear regression fit. Plot showing body mass and generation time for extant species (red symbols), and body mass and imputed generation time for fossil taxa (blue symbols) using a linear regression (OLS) fit. (PDF) [file pone.0068029.s006.pdf]

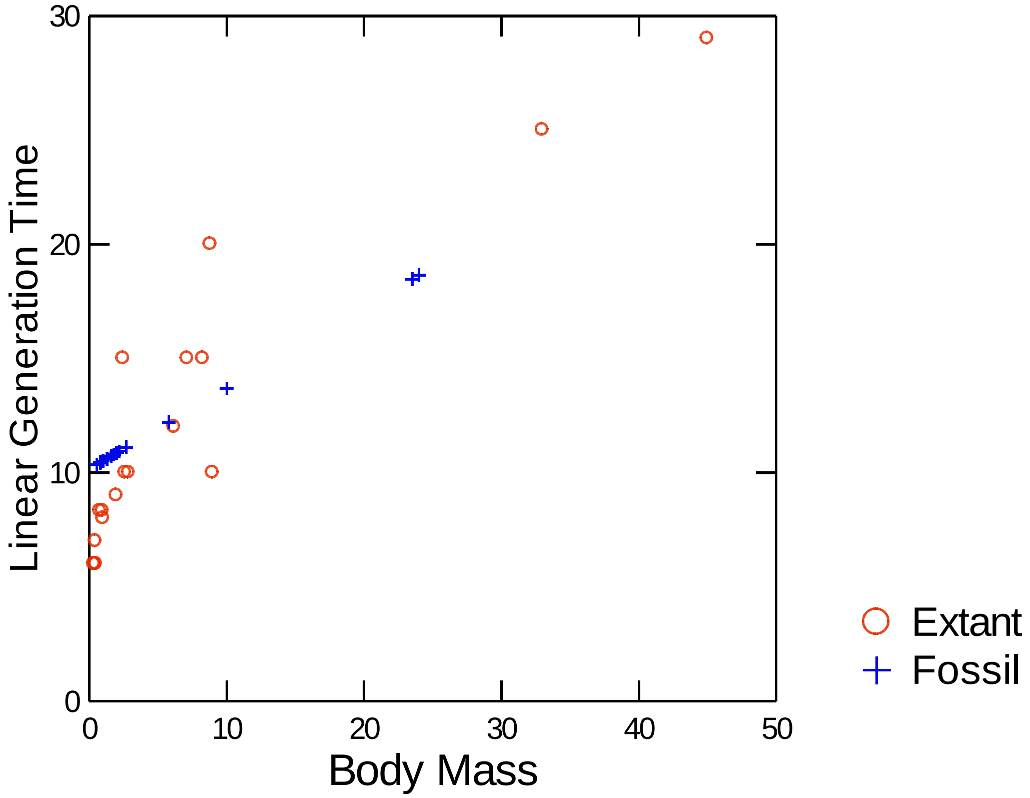

Figure S6 Linear regression fit. Plot showing body mass and generation time for extant species (red symbols), and body mass and imputed generation time for fossil taxa (blue symbols) using a linear regression (OLS) fit.
