# Supplementary material for: Divergence Times and the Evolutionary Radiation of New World Monkeys (Platyrrhini, Primates): An Analysis of Fossil and Molecular Data
Source: PLoS One. 2013 Jun 27;8(6):e68029. doi: 10.1371/journal.pone.0068029 (PMC3694915; doi:10.1371/journal.pone.0068029)
Supplement: Table S1 — OLS results. OLS Regression results for extant taxa. (PDF) [file pone.0068029.s008.pdf]

**Table S1 OLS results.** OLS Regression results for extant taxa

**Regression Coefficients**

| Effect | Coefficient | Standard Error | Std. Coefficient | Tolerance | t | P-value |
|---|---|---|---|---|---|---|
| CONSTANT | 8.559 | 0.799 | 0.000 | . | 10.714 | 0.000 |
| Body Mass | 0.000 | 0.000 | 0.907 | 1000 | 8.613 | 0.000 |

F-ratio: 74.178; P-value: 0.000
R: 0.907; Squared R: 0.823; Adjusted Squared R: 0.811
