# Supplementary material for: Divergence Times and the Evolutionary Radiation of New World Monkeys (Platyrrhini, Primates): An Analysis of Fossil and Molecular Data
Source: PLoS One. 2013 Jun 27;8(6):e68029. doi: 10.1371/journal.pone.0068029 (PMC3694915; doi:10.1371/journal.pone.0068029)
Supplement: Material S1 — Regression between body mass and generation time, imputation procedure and molecular rate correction. (PDF) [file pone.0068029.s009.pdf]

Supplementary Material 1. Regression between body mass and generation time, imputation procedure and molecular rate correction.

We applied a slight modification of the methods recently proposed by Steiper and Seiffert [27] and Langergraber et al. [22] to estimate the initial divergence time of platyrrhines. This method is based on the argument that by estimating an external molecular rate, or the rate at which the nuclear DNA sequence diverged in the genome, DNA differences can be converted into divergence times independently of fossil calibration constraints [22,71]. The only available direct estimation of a molecular rate among primates, independent from fossil constraints, is for the human lineage [22]. Since this rate is not necessarily the same for platyrrhine primates, a procedure to correct for possible differences in substitution rate is needed [27]. Particularly, we employed body size and generation time estimates for extant and fossil platyrrhines to obtain a maximum and minimum corrected substitution rate that is applicable to the different platyrrhine main clades.

First, we applied an imputation procedure [74] for estimating generation time of fossil taxa. Generation time for extant platyrrhine species was obtained from IUCN [78]. By using body size values obtained from Smith and Jungers [79] and Fleagle [7] for extant and fossil taxa, respectively, we applied a linear regression (OLS), a quadratic curve and the EM algorithm [74] and obtained an estimation of generation time for fossil taxa. The OLS results are shown in the Table S1. The linear regression and EM algorithm generated highly biased estimations (see figures S1-S3 below) for both smaller and bigger taxa. For this reason we used the quadratic estimation in the following analyses (Table 5). The generation times for the fossils are in whole numbers because we rounded the estimation after applying the formulas. This is comparable to the whole numbers generally used with extant species.

Second, we used generation times of extant taxa and the imputed generation times for fossil genera to correct the substitutions rate using rule of three [22]. Particularly, we used the mean generation time inferred for each main clade (using extant and fossil taxa) as the best estimation of generation times along their whole evolutionary history [see 85]. This is a different approach to that of Steiper and Seiffert [27]. In this way, the changes in substitution rate along our tree are a function of changes in body size and generation times of the studied primate species, such as it is predicted by the hypothesis that most mutations occur during DNA replication [73].

Finally, after correcting the substitution rates for each main clade, we used these estimations to infer the divergence time for each node of interest (Table 8). To do this, we estimated a Maximum Likelihood tree with a general time reversible substitution model and gamma distribution and then constructed a linearized tree using Mega 5.05 [75,76].
